# Upward synaptic scaling is dependent on neurotransmission rather than spiking

**DOI:** 10.1038/ncomms7339

**Published:** 2015-03-09

**Authors:** Ming-fai Fong, Jonathan P. Newman, Steve M. Potter, Peter Wenner

**Affiliations:** 1 Department of Physiology, Emory University School of Medicine, Atlanta, Georgia 30322, USA; 2 Laboratory for Neuroengineering, Coulter Department of Biomedical Engineering, Georgia Institute of Technology, Atlanta, Georgia 30332, USA; 3 Picower Institute for Learning and Memory, Massachusetts Institute of Technology, Cambridge, Massachusetts 02139, USA

## Abstract

Homeostatic plasticity encompasses a set of mechanisms that are thought to stabilize firing rates in neural circuits. The most widely studied form of homeostatic plasticity is upward synaptic scaling (upscaling), characterized by a multiplicative increase in the strength of excitatory synaptic inputs to a neuron as a compensatory response to chronic reductions in firing rate. While reduced spiking is thought to trigger upscaling, an alternative possibility is that reduced glutamatergic transmission generates this plasticity directly. However, spiking and neurotransmission are tightly coupled, so it has been difficult to determine their independent roles in the scaling process. Here we combined chronic multielectrode recording, closed-loop optogenetic stimulation, and pharmacology to show that reduced glutamatergic transmission directly triggers cell-wide synaptic upscaling. This work highlights the importance of synaptic activity in initiating signalling cascades that mediate upscaling. Moreover, our findings challenge the prevailing view that upscaling functions to homeostatically stabilize firing rates.

## Abstract (web summary)

Synaptic upscaling is characterized by an increase in the strength of excitatory inputs to a neuron as a compensatory response to chronic reductions in spiking activity. Here, the authors demonstrate that reduced glutamatergic transmission, rather than reduced spiking activity, directly triggers upscaling.

## Graphical abstract

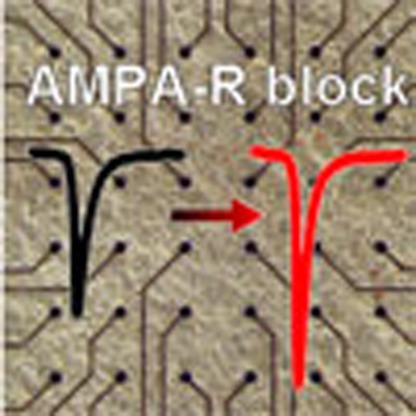

Neural networks need to maintain specific levels and patterns of spiking activity in order to function properly. As aberrant activity patterns often develop following neural injury and disease, it is important to identify the triggers and mechanisms of plasticity that influence neural excitability^1^,^2^,^3^,^4^,^5^,^6^,^7^. Homeostatic plasticity encompasses a set of mechanisms that act to maintain appropriate levels of spiking activity^8^,^9^. The most commonly studied form of homeostatic plasticity is AMPAergic (α-Amino-3-hydroxy-5-methyl-4-isoxazolepropionic acid) synaptic upscaling, a cell-wide multiplicative increase in the quantal amplitudes of spontaneous AMPAergic currents following chronic reductions in firing rate^10^,^11^.

The prevailing view is that upscaling is directly triggered by reductions in firing rate because multiplicative increases in AMPAergic quantal amplitude are observed following chronic blockade of voltage-gated sodium channels^11^,^12^,^13^. However, upscaling is also observed following chronic blockade of AMPA receptors (AMPARs)^11^,^14^,^15^, leaving open the possibility that reduced AMPAergic transmission itself, rather than reduced spiking, could directly trigger upscaling. This possibility has been difficult to test because spiking and AMPAergic transmission are highly coupled processes.

A few studies have circumvented this difficulty by manipulating spiking activity in individual neurons while leaving neurotransmission largely intact. One study blocked voltage-gated sodium channels at the soma, which lead to an accumulation of AMPARs, consistent with upscaling being triggered by reduced spiking^16^. Two other studies chronically hyperpolarized individual neurons by overexpressing a potassium channel; this reduction in spiking did not trigger upscaling^12^,^17^. Therefore, it remains unclear whether a reduction in spiking is sufficient to trigger upscaling without a concomitant reduction in neurotransmission. If reduced spiking can directly trigger scaling, this would provide an elegant mechanism for tuning overall neuronal excitability without disrupting the relative distribution of synaptic strengths imposed by Hebbian mechanisms^18^,^19^. Conversely, if reduced neurotransmission triggers scaling, this would suggest that scaling may be similar to a local form of homeostatic plasticity that acts to stabilize the strength of individual synapses in response to altered synaptic activity^20^,^21^,^22^,^23^. On the basis of the conflicting evidence from studies that have reduced activity in single cells, it has been difficult to deduce the trigger for scaling and its functional significance within a neural circuit.

In the current study, we determine the distinct roles of spiking and neurotransmission as triggers for upscaling by independently manipulating both AMPAergic transmission and spiking activity at the network level. First, we use microelectrode array (MEA) recordings to continuously monitor spiking activity, and show that reductions in spiking do not correlate with the magnitude of upscaling. Next, we show that chronically blocking AMPARs is sufficient to trigger upscaling even when firing rates are clamped to normal levels for the duration of receptor blockade using closed-loop optogenetic control. Finally, we show that scaling induced by blocking spikes is attenuated when we enhance quantal AMPAergic currents. Together, these experiments suggest that cell-wide upscaling is directly triggered by reduced AMPAergic transmission, and not reduced spiking. Our findings challenge the prevailing view that upscaling acts to homeostatically maintain firing rate and suggests that the signalling pathways that mediate scaling are tied to synaptic activity.

## Results

### Spiking persists during chronic AMPAergic blockade

**Figure 1:**
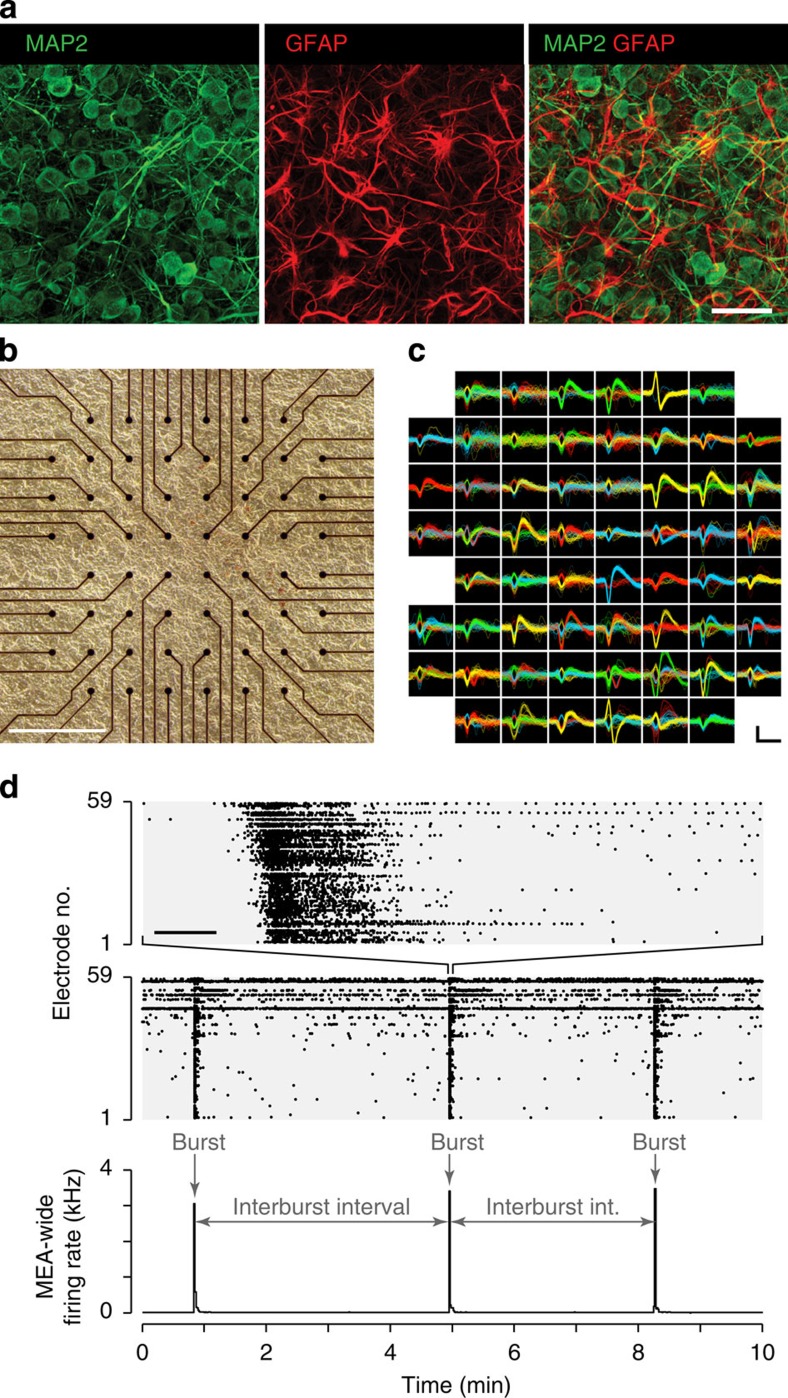
Overview of microelectrode array recordings for monitoring spiking activity. (**a**) Confocal micrograph of dissociated cortical culture containing neurons (MAP2) and astrocytes (GFAP). Scale bar, 50 μm. (**b**) Phase-contrast micrograph of dissociated cortical culture grown on a planar MEA. Scale bar, 500 μm. (**c**) Extracellular spike waveforms recorded on each microelectrode shown in **b**. For each electrode, colours denote different sorted units. Scale bars, 2 ms, 100 μV. (**d**) *Top*, rastergram of spike times occurring during a network-wide burst, typical of dissociated cortical cultures. Scale bar, 200 ms. *Middle*, rastergram showing multiple bursts over several minutes. *Bottom*, time histogram of spikes occurring across the entire MEA over the same time course shown in middle panel. MEA-wide firing rate represents the number of spikes occurring in each bin. Bin size, 1 s.

We sought to examine the nature of spiking activity in a developing neural network, and to assess how this activity is altered during perturbations that trigger synaptic scaling. To this end, we used planar MEAs to record extracellular spiking activity from hundreds of neurons embedded in dissociated cortical cultures of neurons and glia during their second week *in vitro* (Fig. 1a–c). For each culture, the overall firing rate was assessed by summing the spikes recorded across all MEA electrodes, and dividing by the elapsed time interval^24^. Consistent with previous work^24^,^25^,^26^, MEA recordings showed that spikes occurred primarily during network-wide population bursts (hereafter referred to as bursts); however, low levels of asynchronous spiking were also observed during the interburst interval (Fig. 1d).

**Figure 2:**
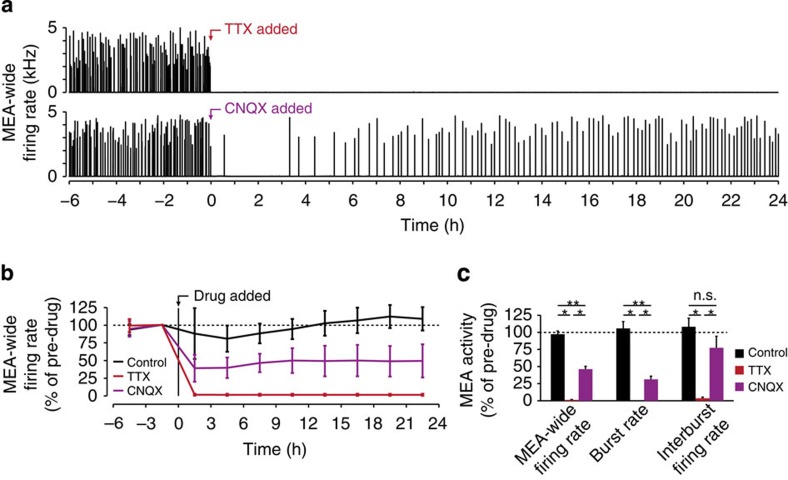
Spiking and bursting persist during AMPAergic transmission blockade. (**a**) MEA-wide firing rate histograms from example recordings before and during application of TTX (1 μM) or CNQX (40 μM). Bin size, 1 s. (**b**) The mean MEA-wide firing rate over time for different conditions (vehicle-treated controls, *n*=12 cultures; TTX, *n*=8 cultures; CNQX, *n*=13 cultures). Values are normalized to the firing rate during the 3-h window before drug/vehicle application. Bin size, 3 h. Error bars, s.d. (**c**) The mean MEA-wide firing rate (control, 97.3±4.6%; TTX, 1.1±0.5%; CNQX, 46.2±4.1%; *P*<10^−6^), burst rate (control, 105.8±10.0%; TTX, 0%; CNQX, 31.2±4.8%; *P*<10^−6^) and interburst firing rate (control, 108.1±12.7%; TTX, 3.6±1.5%; CNQX, 77.4±16.8%; *P*<10^−4^) over the entire 24-h treatment window, normalized to pre-drug values. Nonsignificant differences denoted by n.s. Significant differences denoted by *P<10^−3^, **P<10^−4^. Error bars, s.e.m.

We next examined how a 24-h blockade of either voltage-gated sodium channels or AMPA/kainate receptors modulated spiking and bursting activity. Both perturbations are thought to trigger upscaling by reducing spiking activity. Consistent with our expectations, the voltage-gated sodium channel blocker tetrodotoxin (TTX) eliminated spiking activity for the entire 24-h treatment (Fig. 2a,b). In contrast, the AMPA/kainate receptor antagonist 6-cyano-7-nitroquinoxaline-2,3-dione (CNQX) only partially reduced spiking activity compared with pre-drug levels^15^,^27^,^28^ (Fig. 2a,b). The CNQX-induced reduction in the firing rate was primarily due to a reduction in burst frequency, while spiking during the interburst interval was not significantly affected (Fig. 2c). Spiking and bursting were reduced during the first few hours of the CNQX treatment, but typically began to recover by the end of 24 h (Fig. 2a,b). This recovery was highly variable across cultures (Fig. 2b, Supplementary Fig. 1a–d), and was likely facilitated by NMDAergic (N-methyl-D-aspartate) transmission^27^,^29^ (Supplementary Fig. 2). Notably, any recovery could not have been caused by AMPAergic upscaling since AMPARs were blocked by CNQX. While the CNQX-induced reduction in spiking was variable, some degree of spiking and bursting always persisted, unlike cultures treated with TTX. The distinct effects of TTX and CNQX on spiking activity suggest that they could also have different effects on activity-dependent processes such as synaptic scaling.

### Changes in spiking and synaptic strength not correlated

**Figure 3:**
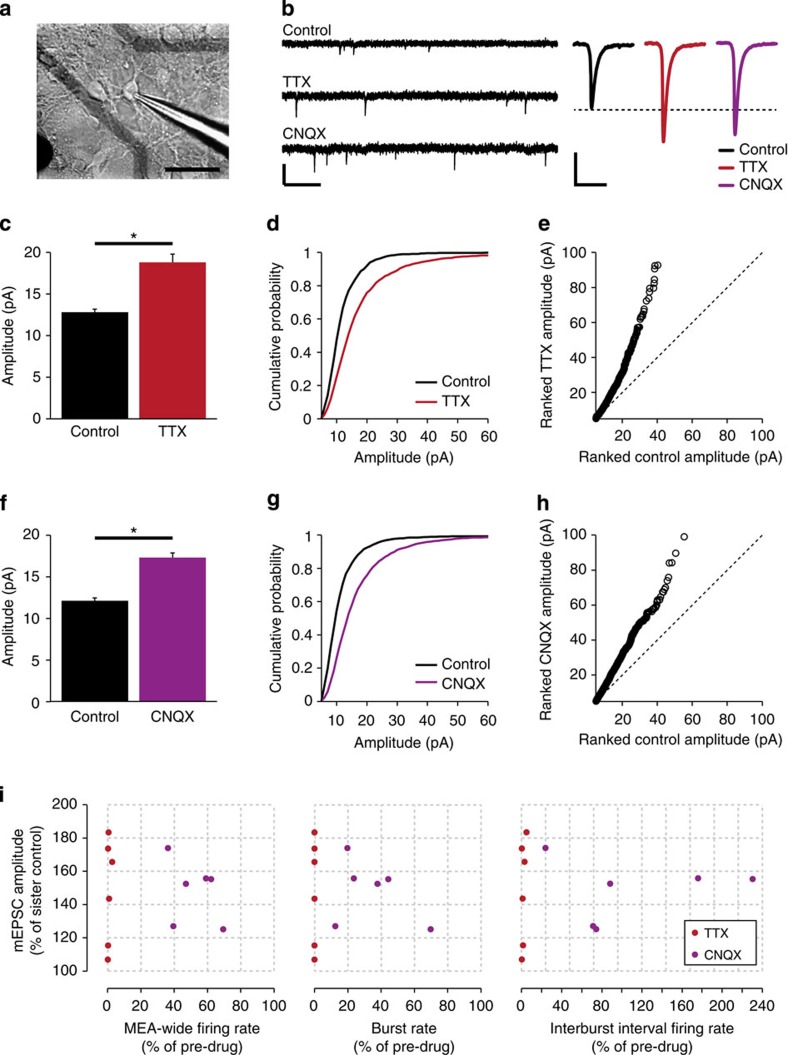
Reductions in spiking are not correlated to the magnitude of synaptic scaling. (**a**) Pyramidal cell during whole-cell recording. Scale bar, 50 μm. (**b**) *Left*, sample mEPSC recordings following 24-h treatment with vehicle, TTX (1 μM) or CNQX (40 μM). Scale bars, 25 pA, 200 ms. *Right*, average mEPSC waveforms. Scale bars, 5 pA, 20 ms. (**c**) The mean mEPSC amplitude from six sister culture pairs (control: 12.8±0.4 pA, *n*=47 cells; TTX: 18.8±1.0 pA, *n*=58 cells; *P*<10^−5^). Error bars, s.e.m. (**d**) Cumulative distribution of mEPSC amplitudes following TTX or vehicle treatment. The multiplicatively scaled TTX distribution matches control (see Supplementary Fig. 3, *P*>0.6). (**e**) Ranked TTX mEPSC amplitudes plotted against ranked control amplitudes (linear fit, *R*^2^=0.975). Dotted line denotes the line of identity. (**f**) The mean mEPSC amplitude for 10 sister culture pairs (control: 12.1±0.3 pA, *n*=89 cells; CNQX, 17.3±0.5 pA, *n*=94 cells; *P*<10^−12^). (**g**) Cumulative distribution of mEPSC amplitudes following CNQX or vehicle treatment. The multiplicatively scaled CNQX distribution matches control (see Supplementary Fig. 3, *P*>0.9). (**h**) Ranked CNQX mEPSC amplitudes plotted against ranked control amplitudes (linear fit, *R*^2^=0.996). Dotted line denotes line of identity. (**i**) *Left*, the mean mEPSC amplitude for individual cultures plotted against the average firing rate they exhibited during the 24-h TTX or CNQX treatment. mEPSC amplitudes are normalized to corresponding sister control cultures, and MEA-recorded activity is normalized to pre-drug levels (linear fit, *r*=−0.047). *Centre and right*, the mean mEPSC amplitude plotted against burst rate and interburst firing rate, respectively (linear fits: burst rate, *r*=−0.114, interburst firing rate, *r*=0.044).

We hypothesized that cultures experiencing greater reductions in the spiking activity would also experience more dramatic upscaling of quantal amplitude. To test this hypothesis, we used whole-cell voltage clamp recordings to measure miniature excitatory postsynaptic currents (mEPSCs) from pyramidal cells following 24-h application of TTX or CNQX (Fig. 3a,b). Consistent with previous studies^11^,^15^, chronic TTX or CNQX treatment produced similar increases in mEPSC amplitude compared with vehicle-treated sister control cultures (Fig. 3b,c,f; TTX, 146.8±8.0% of control; CNQX, 142.9±4.5% of control) without changes in mEPSC frequency (Supplementary Fig. 3). Further, the distributions of mEPSC amplitudes scaled multiplicatively (Fig. 3d,e,g,h, Supplementary Fig. 3). Surprisingly, cells from CNQX-treated cultures, which exhibited only moderate reductions in spiking, scaled equally to cells in TTX-treated cultures that exhibited complete elimination of spiking.

To quantify the relationship between spiking and scaling, we compared changes in spiking and mEPSC amplitude for individual sister culture pairs. For each culture, we compared the reduction in MEA-recorded firing rate during the 24-h TTX or CNQX treatment to the increase in the mean mEPSC amplitude recorded following the treatment (Fig. 3i). While all TTX- and CNQX-treated cultures exhibited reduced firing rates and increases in average mEPSC amplitude, we observed no correlation between the magnitude of the reduction in firing rate and the resultant increase in mEPSC amplitude. In addition, there was no correlation between the increase in the mEPSC amplitude and burst rate or interburst firing rate (Fig. 3i). Moreover, there was no relationship between changes in the mEPSC amplitude and firing rate during the first few hours after TTX or CNQX treatment (Supplementary Fig. 1e). Overall, the lack of correlation between spiking activity and scaling demonstrates that the degree of upscaling cannot be fully explained by changes in spiking activity.

There are several reasons why upscaling might be poorly correlated with changes in spiking activity. It is possible that any reduction in spiking beyond a certain threshold triggers the maximal scaling response. For instance, the rate of insertion of AMPARs that mediates the expression of scaling might saturate, such that further increases in quantal amplitude are not possible during the 24-h treatment period. Alternatively, it is possible that postsynaptic spiking itself does not directly trigger the scaling process and that reductions in AMPAergic transmission might instead directly trigger upscaling.

Blockade of AMPAergic transmission using CNQX reduces excitatory synaptic input to cells, and thus decreases postsynaptic spiking. Conversely, blockade of spiking using TTX eliminates evoked neurotransmitter release, and thus reduces the amount of glutamate that can activate AMPARs. As TTX and CNQX each reduces spiking and AMPAergic transmission either directly or indirectly, identifying their independent effects on the scaling process has proven challenging. To address this, we developed two strategies for examining how chronic reductions in spiking or neurotransmission independently affect synaptic scaling. First, we blocked AMPAergic transmission while maintaining normal spiking activity. Second, we blocked spiking while partially restoring AMPAR activation.

### Optogenetic feedback restores spiking during AMPAR block

**Figure 4:**
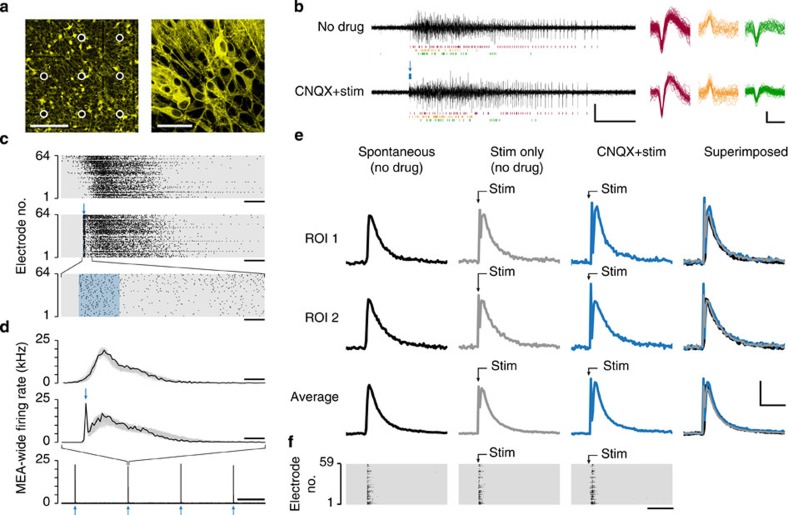
Optogenetic stimulation recreates spontaneous-like bursting during AMPAergic transmission blockade. (**a**) Confocal micrograph of neurons in a cortical culture expressing ChR2-eYFP. Microelectrodes are circled in white. Scale bars, 200 μm (left), 50 μm (right). (**b**) *Left*, voltage traces recorded from a single microelectrode during a spontaneous burst (top) and a photostimulation-evoked burst following the addition of CNQX (40 μM, bottom). The blue arrow denotes the timing of the light pulse stimulation and the blue rectangle indicates the pulse duration (10 ms). The rastergrams (coloured vertical bars) below each voltage trace denote the spike times of three different extracellular units captured on the electrode. *Right*, expanded voltage traces showing all spikes detected during burst (separate units displayed in different colours). Scale bars, 50 μV, 200 ms (left); 25 μV, 1 ms (right). (**c**) Rastergrams showing spike times recorded across all electrodes during a spontaneous burst (top) and an optically evoked burst after the addition of CNQX (middle). The recruitment of spikes across the MEA is similar between the two conditions. Blue arrow denotes the timing of the light pulse. An expanded rastergram shows spikes occurring at burst onset (bottom) and blue shading denotes when light is on. Scale bars, 100 ms (top and middle), 5 ms (bottom). (**d**) MEA-wide firing rate computed during bursts shown in **c**, denoted by black lines. All bursts occurring during the 3-h pre-drug condition (top) and 24-h CNQX with photostimulation condition (middle) are plotted in grey. Zooming out in time (bottom) shows that each stimulus (blue arrows) reliably evokes a burst. Bin size, 10 ms. Scale bars, 100 ms (top and middle), 2 min (bottom). (**e**) Calcium transients (▵*F*/*F*) for two different ROIs and a 10-ROI average are shown during a spontaneous burst, an optically-evoked burst, and an optically evoked burst in the presence of CNQX. All traces were taken from the same culture. Fast calcium transient immediately follows the 10-ms pulse of blue light stimulation (direct illumination through filters, not dependent on calcium indicator). Scale bars, 2 s, 40% Δ*F*/*F*. (**f**) Rastergrams showing spike times for all MEA electrodes recorded concurrently with calcium transients shown in **e**. Scale bar, 2 s.

**Figure 5:**
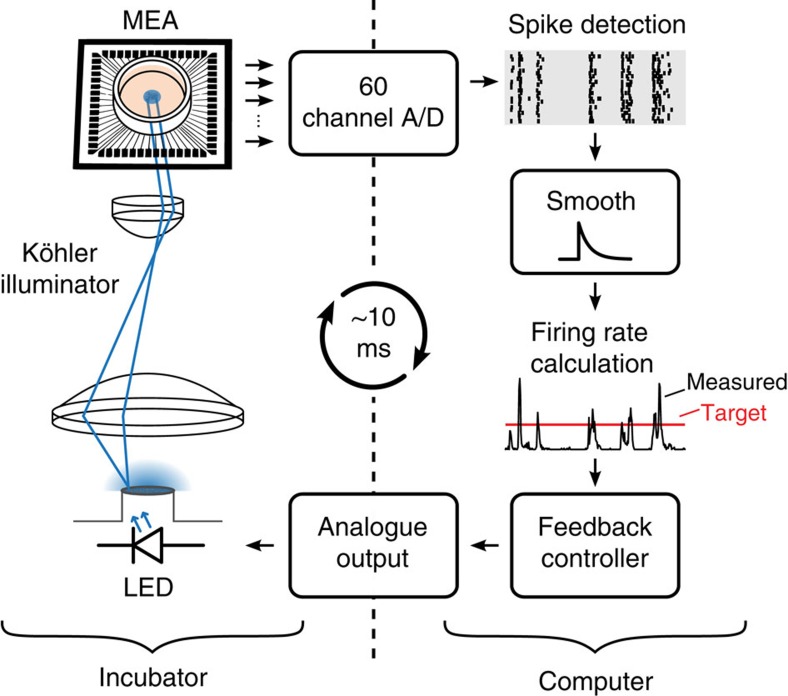
Schematic of closed-loop optical stimulation system. Spiking activity is recorded through the MEA. When the integrated error between the target and measured MEA-wide firing rate becomes positive, a 10-ms current pulse is delivered to a blue LED. A Köhler illuminator is used to produce uniformly bright illumination at the cell layer.

In order to examine the effects of reducing AMPAergic transmission independent of spiking, we developed a closed-loop optical stimulation system for restoring normal levels of spiking activity during chronic CNQX treatment. We used an adenoassociated virus to infect cells with the channelrhodopsin-2 gene (ChR2; H134R mutant^30^) in cultured neurons, and observed expression throughout the culture within a week (Fig. 4a). To deliver optical stimuli, we used a custom light-emitting diode (LED) stimulator (see Methods). Blue light (465 nm) was passed through a randomized fibre bundle and fed to a custom optical train, providing uniformly distributed illumination in the plane of the culture (Fig. 5, left; 10.1 mW mm^−2^).

Because the reductions in firing rate that accompany CNQX application were primarily because of reductions in network-wide bursts (Fig. 2c), we used a stimulation strategy that reinstated bursts. A 10-ms pulse of blue light reliably evoked short-latency spikes that resulted directly from ChR2 activation, followed by a longer latency barrage of action potentials. These longer latency barrages of spikes, which occurred tens to hundreds of milliseconds after the blue light pulse terminated, closely resembled spontaneously occurring bursts in terms of time course and profile of network recruitment (Fig. 4b–d; Supplementary Figs 4,5). We suspect that these bursts are dependent on NMDAergic transmission, since they could not be reproduced during NMDA receptor blockade (Supplementary Fig. 6).

Previous work suggests that reductions in somatic calcium influx are critical to the upscaling process^16^. Therefore, we measured somatic calcium transients that occurred during spontaneous bursts using the calcium-sensitive indicator Rhod-3 (see Methods), and compared these with calcium transients that occurred during optically evoked bursts in the presence of CNQX. Under both conditions, the vast majority of somatic calcium transients were time-locked to MEA-recorded bursts (Fig. 4e,f) and occurred across the Rhod3-labelled neuronal population (Supplementary Movie 1). In addition, the profile of calcium influx during optically evoked bursts in the presence of CNQX resembled that of spontaneously occurring bursts (Fig. 4e,f, Supplementary Fig. 7, Supplementary Movie 1). Together, the results suggest that our blue light stimulus could reliably produce bursts during AMPAergic blockade that were similar to spontaneous bursts, both in spike timing and in somatic calcium entry.

**Figure 6:**
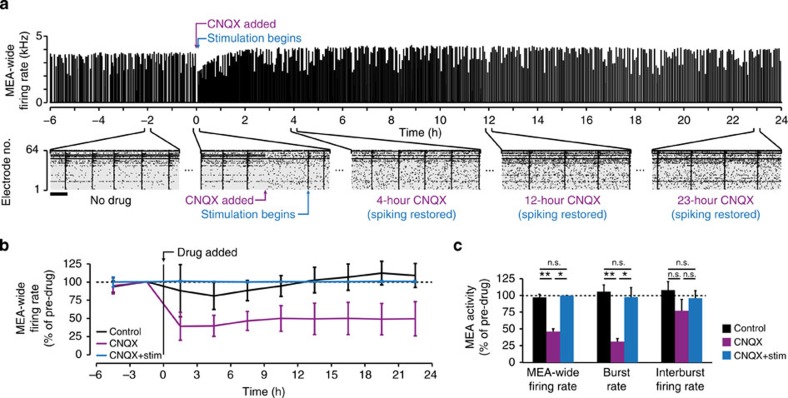
Closed-loop optical stimulation restores spiking activity and calcium transients following AMPAergic transmission blockade. (**a**) *Top*, MEA-wide firing rate from example recording before and during application of CNQX (40 μM), with the pre-CNQX firing rate restored using closed-loop photostimulation. The closed-loop controller begins 5 min after CNQX is added to verify that the drug has taken effect. Bin size, 1 s. *Bottom*, rastergrams show 15-min segments of spiking activity at different time points throughout the recording. Neurons throughout the culture contribute to restored spiking activity during the entire 24-h CNQX treatment. Scale bar, 2 min. (**b**) The mean MEA-wide firing rate over time for CNQX-treated cultures with restored spiking (*n*=5 cultures). Control and CNQX values from Fig. 2b are shown for comparison (controls, *n*=12 cultures; CNQX, *n*=13 cultures). Closed-loop stimulation effectively locked firing rate to pre-CNQX levels. Bin size, 3 h. Error bars, s.d. (**c**) The mean MEA-wide firing rate, burst rate and interburst firing rate for CNQX+photostimulation cultures over the 24-h treatment window, with control and CNQX values from Fig. 2c shown for comparison. CNQX-treated cultures with restored spiking showed no differences in firing rate or burst rate compared with vehicle-treated controls (MEA-wide firing rate, 100.2±0.4%, *P*<0.6; burst rate, 97.7±32.0%, *P*<0.9; interburst firing rate, 96.2±24.9%, *P*<0.9). Nonsignificant differences denoted by n.s. Significant differences denoted by *P<10^−3^, **P<10^−4^.

To achieve precise control of the MEA-wide firing rate, we delivered optical stimulation in real-time based on spiking activity recorded through the MEA^31^. The MEA-wide firing rate was calculated every 10 ms, and a brief pulse (10 ms) of blue light was delivered if the integrated error between the target and measured firing rate became positive (Fig. 5, methods). In order to restore normal levels of spiking during an AMPAR blockade, we treated cultures with CNQX and began closed-loop optical stimulation with a target spiking level set to the pre-drug firing rate (Fig. 6a). Closed-loop optical stimulation effectively restored the pre-drug firing rate throughout CNQX application (Fig. 6b) while preserving spiking correlations between electrodes (Supplementary Fig. 5). Further, our controller effectively restored burst rate (Fig. 6c) and burst shape (Supplementary Fig. 4).

### Reduced spiking is not required to trigger upward scaling

**Figure 7:**
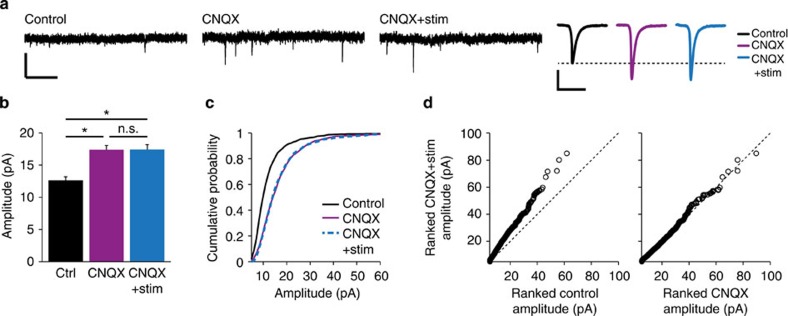
Reduced AMPAergic transmission directly triggers upward synaptic scaling. (**a**) *Left*, sample mEPSC recordings following 24-h treatment with vehicle, CNQX (40 μM) or CNQX+photostimulation. Scale bars, 25 pA, 200 ms. *Right*, average mEPSC waveforms. Scale bars, 5 pA, 20 ms. (**b**) The mean mEPSC amplitude for five sister culture pairs from the three treatment conditions (control: 12.6±0.6 pA, *n*=44 cells; CNQX: 17.4±0.7 pA, *n*=51 cells; CNQX+photostimulation: 17.4±0.8 pA, *n*=46 cells; *P*<10^−6^). Nonsignificant differences denoted by n.s. Significant differences denoted by *P<10^−5^. Error bars, s.e.m. (**c**) Cumulative distribution of mEPSC amplitudes following the three treatment conditions. Multiplicatively scaled CNQX and CNQX+photostimulation distributions matched control (*P*>0.9 for both, Supplementary Fig. 3a), and there was no difference between the CNQX and CNQX+photostimulation distributions (*P*>0.9). (**d**) Ranked CNQX+photostimulation mEPSC amplitudes plotted against ranked control or CNQX amplitudes (linear fits, *R*^2^=0.998 and *R*^2^=0.995, respectively). Dotted line denotes the line of identity.

Having reinstated the pre-drug firing rate during AMPAergic transmission blockade (Fig. 6), we next examined whether this restored spiking activity would prevent upward synaptic scaling. To this end, we recorded mEPSCs from triplicate sister cultures: [1] vehicle-treated control cultures experiencing normal AMPAergic transmission and normal spiking activity, [2] CNQX-treated cultures experiencing no AMPAergic transmission and reduced spiking and [3] photostimulated CNQX-treated cultures experiencing no AMPAergic transmission but restored spiking activity (Fig. 7a). In line with previous observations^11^,^14^,^15^ (Fig. 3), mEPSC amplitudes from CNQX-treated cultures exhibiting reduced spiking were scaled up as compared with sister control cultures (Fig. 7, Supplementary Fig. 3a). Interestingly, mEPSC amplitudes from CNQX-treated cultures with restored spiking also scaled up (Fig. 7, Supplementary Fig. 3a), and the mean mEPSC frequency was increased over control values (*P*<10^−2^; Supplementary Fig. 3b). The distributions of mEPSC amplitudes from CNQX-treated cultures experiencing restored versus reduced activity were statistically indistinguishable (Fig. 7c,d; *P*>0.9). These results demonstrate that reductions in spiking are not required to trigger upward synaptic scaling. Instead, reduced AMPAergic transmission can directly and independently trigger upscaling when spiking and the associated somatic calcium transients are restored.

### Reduced AMPAR activation triggers upward synaptic scaling

Because reduced AMPAergic transmission can directly trigger upscaling, we hypothesized that chronic blockade of the spiking activity (TTX application) leads to upscaling by preventing spike-dependent release of neurotransmitter and consequent reductions in AMPAergic transmission. Alternatively, it is possible that either a reduction in spiking or a reduction in AMPAergic transmission could independently trigger scaling.

**Figure 8:**
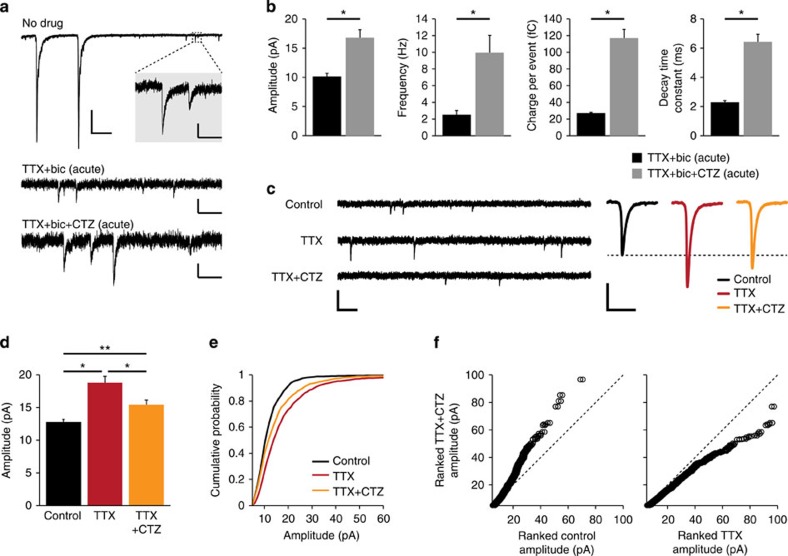
Upscaling that follows chronic spiking blockade is mediated by reduced AMPAR activation. (**a**) *Top*, sample postsynaptic currents recorded before any drugs are added. Scale bars, 200 pA, 1 s. The shaded inset shows lower-amplitude events. Scale bars, 10 pA, 100 ms. *Middle and bottom*, sample AMPAergic mEPSCs recorded before (middle) and after (bottom) the addition of CTZ (100 μM). Scale bars, 10 pA, 100 ms. (**b**) The mean amplitude (*P*<10^−3^), frequency (*P*<10^−2^), charge per event (*P*<10^−7^) and decay time constant (*P*<10^−6^) of AMPAergic mEPSCs before and during acute application of CTZ (before, *n*=10 cells; during, *n*=11 cells). Significant differences denoted by asterisks. Error bars, s.e.m. (**c**) *Left*, sample mEPSC recordings following 24-h treatment with vehicle, TTX (1 μM) or TTX+CTZ. Scale bars, 25 pA, 200 ms. *Right*, average mEPSC waveforms. Scale bars, 5 pA, 20 ms. (**d**) The mean mEPSC amplitude for six sister culture pairs treated from the three treatment conditions (control and TTX cultures same as Fig. 3c; TTX+CTZ: 15.5±0.7 pA, *n*=50 cells; *P*<10^−5^). Significant differences denoted by *P<10^−2^ and **P<10^−5^. Error bars, s.e.m. (**e**) Cumulative distribution of mEPSC amplitudes following the three treatment conditions (control and TTX cultures same as Fig. 3d). The distribution of mEPSC amplitudes is significantly different between the TTX and TTX+CTZ conditions (*P*<10^−6^). (**f**) Ranked TTX+CTZ mEPSC amplitudes plotted against ranked control or TTX amplitudes (linear fits, *R*^2^=0.990 and *R*^2^=0.989, respectively). Dotted line denotes the line of identity.

To test the importance of reduced AMPAergic transmission on TTX-induced synaptic scaling, we sought to enhance the quantal AMPAergic currents that remained during a spike blockade. AMPARs mediate fast glutamatergic transmission and desensitize quickly after binding glutamate. In order to enhance AMPAergic mEPSCs we used cyclothiazide (CTZ), an AMPAR modulator known to increase receptor open time^32^, as well as spontaneous presynaptic release^33^. In the presence of TTX, the amplitude and frequency of AMPAergic mEPSCs was significantly increased when CTZ was added (Fig. 8a,b), and this effect persisted for at least 11 h of drug application (Supplementary Fig. 8).

We also characterized how somatic calcium transients were affected by TTX alone versus TTX and CTZ treatments. Whereas untreated cultures exhibited synchronous somatic calcium transients across many neurons during spontaneous MEA-recorded bursts, synchronous transients were eliminated in the presence of TTX, regardless of whether CTZ was also present (Supplementary Fig. 9). Instead, there were smaller asynchronous calcium transients observed in TTX-treated cultures, which did not co-localize with the somata of bursting neurons. These asynchronous transients became more prominent when quantal AMPAergic currents were enhanced with CTZ (Supplementary Movie 2; 31 active regions in TTX and 93 active regions in TTX+CTZ across five cultures observed for 3 min in each condition). The small asynchronous calcium transients could be occurring in dendrites or in glia, as both dendritic processes and astrocytes were dense throughout our cultures (Fig. 1a).

In order to test how partially restoring AMPAergic transmission affected TTX-induced scaling, we treated sister cultures with either TTX alone, TTX and CTZ, or vehicle. As with previous experiments, we recorded spiking activity using a MEA during the 24-h treatment period and recorded mEPSCs from triplicate sister cultures after washing out the drugs. Similar to TTX treatment, co-treatment with TTX and CTZ completely abolished spiking and bursting activity (Supplementary Fig. 10). mEPSC amplitudes from cultures co-treated with TTX and CTZ were significantly reduced compared with sister cultures treated with TTX alone (Fig. 8c,d). On the other hand, mEPSC amplitudes from cultures co-treated with TTX and CTZ were significantly increased compared with vehicle-treated controls. This intermediate increase in synaptic strength is likely because CTZ only increased quantal currents and did not fully restore normal postsynaptic currents observed in control cultures with intact spiking (Fig. 8a, top trace). A multiplicative relationship between mEPSC amplitude distributions existed for all three conditions (Fig. 8e,f, Supplementary Fig. 3a), indicating that the increases in synaptic strength were consistent with synaptic scaling. These results show that partially restoring AMPAR activation during spike blockade can significantly attenuate TTX-induced synaptic scaling. This demonstrates that reductions in AMPAergic transmission are critical for triggering TTX-induced upscaling, and suggests that this form of scaling is similar to that observed following chronic CNQX treatment. Together, our results provide strong evidence that reductions in AMPAR activation in cortical networks trigger compensatory increases in synaptic strength.

## Discussion

The induction of cell-wide homeostatic synaptic scaling is thought to be caused by chronic changes in the firing rate. Our study challenges this basic idea and suggests an alternative trigger for synaptic upscaling. First, we found that reductions in spiking activity were not correlated with the magnitude of upscaling. We then showed that reductions in AMPAR activation alone were sufficient to elicit upward synaptic scaling. Lastly, we found that increasing AMPAR activation during spike blockade attenuated upscaling. These findings challenge several well-known computational and experimental models of synaptic scaling and have important implications for compensatory plasticity in the context of learning, memory, development and disease.

Synaptic scaling rules were originally proposed as a mathematically tractable mechanism to curb the unbounded synaptic strengthening or weakening predicted by models of Hebbian learning^18^,^19^,^34^,^35^. Since then, there has been a wealth of evidence in support of both upward and downward scaling across a range of experimental contexts^8^,^36^. While previous studies hypothesized that scaling could be triggered by changes in the firing rate, the dependence of scaling on spiking appears to be more nuanced.

The prevailing model of upscaling suggests that chronic reductions in the frequency of somatic action potentials lead to reduced calcium influx, which then triggers upscaling of AMPAergic currents^10^,^16^. However, this model is not compatible with our results. Rather, we found that there was a poor correlation between reductions in the firing rate and the average increase in quantal amplitude (Fig. 3i). Further, in the presence of CNQX, closed-loop optogenetic restoration of the firing rate did not attenuate AMPAergic upscaling (Fig. 7). In addition, our stimulation paradigm effectively restored burst rate (Fig. 6c), burst shape (Fig. 4d) and somatic calcium transients (Fig. 4e,f, Supplementary Fig. 7, Supplementary Movie 1 (refs 26, 37, 38)) during AMPAergic blockade. Together, these results suggest that reductions in spiking or spike-dependent calcium influx are not required to induce upscaling.

Previous studies have examined the consequences of altering spiking in individual neurons while leaving synaptic transmission onto these cells intact. In one set of studies, spiking was chronically reduced in individual postsynaptic cells by overexpression of an inwardly rectifying potassium channel (Kir2.1), *in vitro*^12^ and *in vivo*^17^. The Kir2.1-expressing cells homeostatically recovered spiking activity over a few days, but did not express upscaling, indicating that reductions in spiking alone did not trigger scaling. Our finding that reduced neurotransmission triggers upscaling is consistent with these studies as neurotransmission onto the Kir2.1-expressing cells was unchanged. Another study optogenetically increased spiking in individual neurons, which led to a reduction in mEPSC amplitude, suggesting that increases in spiking alone could trigger downscaling^39^. However, this could be explained by the fact that there are differences in the molecular mechanisms underlying upscaling and downscaling^13^,^40^,^41^,^42^. On the other hand, a separate study puffed TTX locally on the somata of individual neurons in order to block somatic spiking while leaving dendritic neurotransmission intact, and observed accumulation of AMPARs. This suggested that upscaling was directly engaged by reduced somatic spiking^16^, which stands in contrast to the results of our study. While it is unclear precisely why the findings of this study differ from ours, several important differences exist between the two studies, which might contribute to the discrepancy. First, we blocked AMPAergic transmission while maintaining spiking throughout the entire network, rather than maintaining neurotransmission while blocking spiking in individual neurons. Second, we assessed synaptic strength functionally through mEPSC amplitude measurements after 24-h perturbations, rather than by GluA2 fluorescence during the first few hours of the perturbation. Finally, we chronically and continuously monitored spiking and bursting activity before and during each perturbation. Therefore, it is possible that cultures used in these two studies differ in terms of baseline activity levels and firing patterns that were not monitored in the previous study, which may have influenced subsequent plasticity.

While it is possible that upscaling is triggered by alterations in cellular spiking other than firing rates (for example, temporal firing patterns), we find this unlikely. In the aforementioned Kir2.1 transfection studies, dramatic reductions in the firing rate and altered spike timing in individual cells did not trigger upscaling^12^,^17^. Similarly, significant alterations in spiking occur during chronic NMDA receptor blockade^27^,^29^ (Supplementary Fig. 2); however, this perturbation does not appear to trigger upscaling^11^,^20^. Finally, the activity evoked during our photostimulation experiments mimicked spontaneous activity in terms of somatic calcium transients and higher order characteristics of spiking (Supplementary Figs 4–5,7, Supplementary Movie 1), suggesting that cellular spiking patterns were preserved during closed-loop control of network firing rate.

Rather than a spike-dependent model of scaling, our photostimulation experiments support a model where reduced AMPAR activation, independent of changes in spiking activity, triggers upscaling. Further, our CTZ-based experiments demonstrated that TTX-induced scaling is attenuated when spontaneous AMPAergic currents are pharmacologically enhanced. By separately manipulating spiking or AMPAergic transmission throughout the network, we have demonstrated a critical role for glutamatergic transmission, as opposed to spiking, in the induction of upscaling.

While the most parsimonious explanation of the results might be that reducing AMPAR activation at neuronal synapses triggers upscaling, our experiments alter AMPAR activation throughout the culture. Therefore, it is possible that the critical AMPARs are on glia. Indeed, the capacity for glia to sense ambient glutamate has been implicated in the upscaling process^13^. Our cultures have a significant astrocytic population (Fig. 1a) that is well positioned to monitor synaptic activity throughout the network.

Upscaling is characterized by a coordinated increase in the strength of all synaptic inputs onto a neuron. In contrast, recent work has identified a local form of homeostatic plasticity that regulates the strength of individual synapses. In these studies, neurotransmission was reduced in a subset of presynaptic inputs through local application of a receptor antagonist^20^, presynaptic overexpression of Kir2.1 (refs 21, 22) or altering sensory input *in vivo*^23^ (for reviews see refs 43, 44, 45)). Reducing neurotransmission at specific postsynaptic sites (leaving postsynaptic spiking largely intact) resulted in compensatory strengthening of only those synapses. Our finding that cell-wide upscaling is directly triggered by reduced AMPAR activation, rather than reduced firing rate, suggests that the this local compensatory plasticity may be more closely related to cell-wide scaling than previously thought. Specifically, both upscaling and local synaptic compensations are engaged by altered synaptic transmission independent of changes in spiking. Given this similarity, upscaling produced by chronic TTX or CNQX treatment might be the result of bath application of the drugs, which in turn reduce AMPAergic transmission at all synapses and trigger local synaptic compensations throughout the cell. Local strengthening of all synapses on a neuron would then resemble a cell-wide increase in synaptic strength. If upscaling is a cell-wide manifestation of a plasticity that acts to maintain synaptic strength in a synapse-specific manner, then this would represent a very different functional goal than homeostatic maintenance of cellular firing rate.

The possibility that upscaling represents a synapse-specific process raises questions about its relationship to Hebbian forms of plasticity, which is also synapse-specific and widely believed to underlie memory encoding and storage^46^. The cell-autonomous model of synaptic scaling provides an elegant way to stabilize a neuron’s activity levels without disrupting the relative synaptic strengths established through competition-based Hebbian plasticity^18^. Conversely, a synapse-specific model (where scaling emerges when activity at all synapses is similarly disrupted) suggests that information encoded by the relative weights of individual synapses is vulnerable to interference from homeostatic modifications. However, there may be substantial benefits in the interactions of these seemingly antagonistic forms of synaptic plasticity^47^.

## Methods

### Cell culture

Sterilized MEAs (Multichannel Systems, 60MEA200/30iR-Ti-pr) and glass bottom dishes (GBDs; Mattek, P35G-1.5-10-C) were coated with polyethyleneimine (Sigma, P-3143) and laminin (Sigma, L-2020). Neocortical hemispheres were isolated from Sprague–Dawley rats on embryonic day 18 (E18), or equivalent tissue was purchased from BrainBits, LLC (part number: cx). Tissue was enzymatically dissociated using 20 U ml^−1^ activated papain (Roche, 10108014001) at 36.5 °C, mechanically dissociated by trituration, and stained to assess viability using Trypan Blue (Invitrogen, 15250). The resulting cell suspension was diluted to 2,500 live cells·per μl, and 35,000 cells were plated as a 2-mm-diameter drop over the centre of the grid of MEA electrodes or on the GBD culturing surface. Growth medium was modified from Jimbo *et al*.^48^, and it contained: 90% high-glucose DMEM, 10% horse serum, 0.5 mM GlutaMAX, 1 mM sodium pyruvate, 2.5 μg ml^−2^ insulin (pH 7.2, 315 mOsm); no antibiotics or antimycotics were used. The medium was fully exchanged at 1 day *in vitro* (DIV), and half the medium was exchanged every 3 days thereafter. MEAs and GBDs were sealed with fluorinated ethylene-propylene^49^ or polydimethylsiloxane^50^ membranes. Cultures were maintained in an incubator regulated at 35 °C, 5% CO_2_ and 65% relative humidity. Further details of our culturing procedures are described in ref. 51. Sample size was based on number of cultures (always greater than 3). All protocols were in compliance with the National Research Council’s Guide for the care and use of laboratory animals using a protocol approved by the Georgia Tech Institutional Animal Care and Use Committee.

### Pharmacology

Drugs were used in the following concentrations (in μM): TTX, 1; CNQX, 40; bicuculline, 20; CTZ, 100; and APV, 50. CTZ was obtained from ENZO. All other drugs were obtained from Sigma. The vehicle used to treat control cultures was DMSO or water, depending on the solvent used to dilute drug in the experimental group of comparison.

### Transfections

AAV9-hSynapsin-ChR2(H134R)-eYFP was produced by the University of Pennsylvania Vector Core using DNA from Dr. Karl Deisseroth. All cultures used in ChR2 experiments, including controls, were transfected at 1 DIV. The genomic titre was 1 × 10^13^ c.f.u.·ml^−1^, and 0.5 μl was added to 1 ml growth medium at 1 DIV during the first medium exchange. Expression of the eYFP reporter protein was verified during the first week *in vitro* using a confocal microscope (Zeiss LSM 700, Fig. 4a).

### MEA recordings

Experiments began at 8–12 DIVs. MEA recordings were performed in the standard growth medium in a cell culture incubator (35 °C, 5% CO_2_, 65% relative humidity). Voltages recorded through microelectrodes were amplified and bandpass-filtered from 1 Hz to 5 kHz using a 60-channel analogue amplifier (Multichannel Systems, MEA60-Up) and digitized at 25 kHz using the Neurorighter acquisition system^31^,^52^. Voltage recordings were digitally filtered with a third-order Butterworth bandpass from 200–3,000 Hz, and action potentials were detected at threshold of ±5 times the root mean square noise. Offline analyses of the recorded spike data was performed in MATLAB (The Mathworks). The pre-drug period was defined as a 3-h segment preceding drug or vehicle application. The treatment period was defined as the entire 24-h segment during drug or vehicle application. After the treatment period, cultures were washed four times with standard growth medium. Statistical significance for firing and burst rate data was determined using a Kruskal–Wallis test followed by Wilcoxon rank-sum tests with Bonferroni correction for multiple comparisons.

### Whole-cell recordings

mEPSCs were recorded from pyramidal-shaped cells in a continuous perfusion of artificial cerebrospinal fluid (aCSF) containing (in mM): 126 NaCl, 3 KCl, 2 CaCl_2_, 1.5 MgSO_4_, 1 NaH_2_PO_4_, 25 NaHCO_3_ and 25 D-glucose, and saturated with 95% O_2_ and 5% CO_2_ (pH 7.4). To isolate AMPAergic mEPSCs, the external solution was supplemented with 1 μM TTX and 20 μM bicuculline. The solution temperature was regulated at 35 °C using an inline heater (Warner 64-0102). Internal solution contained (in mM) the following: 100 K-gluconate, 30 KCl, 10 HEPES, 2 MgSO_4_, 0.5 EGTA and 3 ATP (pH 7.4). mEPSCs were recorded using an EPC8 amplifier (HEKA). Pipette resistances ranged from 2 to 8 MΩ. mEPSCs were analysed, blind to the treatment condition, using MiniAnalysis (Synaptosoft) and mEPSCs with amplitudes less than 5 pA were excluded from analysis. Statistical significance for mEPSC data was determined using a one-way analysis of variance followed by *t*-tests with Bonferroni correction for multiple comparisons. mEPSC amplitude distributions were compared using the Kolmogorov–Smirnov test. mEPSC characteristics are summarized in Supplementary Fig. 3.

### Optical stimulation

To deliver optical stimuli, a custom current source (http://www.open-ephys.org/cyclops/) was used to drive a blue LED (465±11 nm full-width at half-maximum; LEDEngin, LZ4-00B200). The LED was butt-coupled to a randomized fibre bundle (Schott AG, A21045), which fed light to a custom Köhler illumination train mounted beneath the MEA amplifier. The average network firing rate was calculated every d*t*=10 ms according to




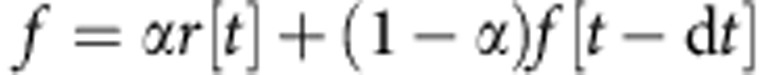




where




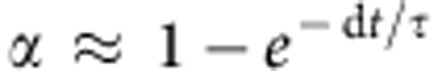




defines a first-order averaging filter with a *τ*=2.5-s time constant and *f*[*t*]=(number of detected spikes in time window d*t*)/d*t* is the instantaneous firing at time *t*. The target rate, f*, was defined as *f*[*t*] over a 3-h period before CNQX application. Five minutes following the application of CNQX to the culturing medium, an error signal was generated between the target and measured firing rate according to




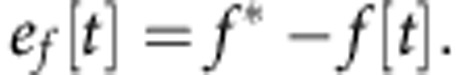




Finally, an on–off controller was used to determine stimulus application according to




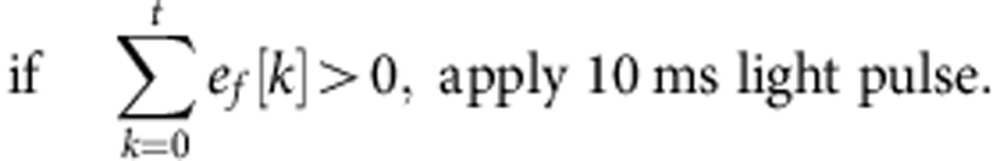




Each stimulus pulse resulted in a uniformly distributed ~10-mW mm^−2^ light in the plane of the culture. The rise and fall times of each LED pulse were ~10 μs.

### Calcium imaging

Cells were loaded with the red-shifted calcium indicator, Rhod-3 AM (Life Technologies R10145) and incubated according to the manufacturer’s instructions, and then rinsed with aCSF. Cytosolic Rhod-3 was imaged on an upright light microscope (Zeiss Axioscope 2) using illumination from a white LED filtered at 540–565 nm (Chroma T565lpxr) and focused through the objective on the specimen. Emitted light was filtered at 570–650 nm (Chroma 41007a) and collected using an EMCCD camera (Photometrics QuantEM:512SC), and images were acquired using Micro-Manager^53^. Blue light pulses (10 ms, 10.1 mW mm^−2^) were delivered using a current controller (Prizmatix TLCC-01) to drive a blue LED, filtered at 460–500 nm (Omega 540DRLP) and focused through the objective on the specimen. Concurrent MEA recording using MC Rack (Multichannel Systems) was performed during all imaging sessions. Recording, imaging and stimulation were synced using MC Stim (Multichannel Systems).

Optical analysis was carried out in order to compare calcium transients during bursts before and after addition of CNQX. Ten regions of interest (ROIs) were drawn around somatic-shaped regions that showed a clear increase in fluorescence during a spontaneous burst and overlapped large neuronal cell bodies, as determined through hSynapsin-driven eYFP labelling. ROIs were used to measure the raw fluorescence, which was then converted to ▵*F*/*F* (raw fluorescence minus a 30-frame average taken before the burst divided by 30-frame average before the burst). For each ROI, ▵*F*/*F* was measured and compared during spontaneous bursts before the CNQX application and during optically evoked bursts in the presence of CNQX. Averages of four to six bursts for each condition were compared using a Wilcoxon rank-sum text. Calcium imaging analysis for CTZ experiments was carried out similarly. Ten ROIs were drawn on the basis of calcium transients of spontaneous bursts and ▵*F*/*F* was measured for 5 min in control conditions, and in the presence of TTX, or TTX and CTZ.

### Immunocytochemistry

Cultures were rinsed with PBS and fixed in 4% paraformaldehyde (pH, 7.2) for 20 min. Fixed cultures were rinsed with PBS and blocked/permeabilized with 10% goat serum and 0.1% Triton-X for 1 h. Cultures were incubated with the primary antibody in 10% goat serum and 0.05% Triton-X for 24 h at 4 °C. Cultures were then incubated with a secondary antibody for 1.5 h. Imaging was performed on a confocal microscope (Zeiss LSM 700). Unless otherwise noted, all procedures were performed at room temperature and cultures were rinsed several times with PBS between steps. Antibody concentrations were as follows: rabbit anti-GFAP (Accurate Chemical, BMDV2023), 1:200; mouse anti-MAP2 (Millipore), 1:300; Alexafluor 594 anti-rabbit (Life Technologies), 1:200; and Alexafluor 488 anti-mouse (Life Technologies), 1:200.

## Additional information

**How to cite this article:** Fong, M.-f. *et al*. Upward synaptic scaling is dependent on neurotransmission rather than spiking. *Nat. Commun.* 6:6339 doi: 10.1038/ncomms7339 (2015).

## Supplementary Material

Supplementary Figures and References Supplementary Figures 1-10 and Supplementary References

Supplementary Movie 1 Calcium transients are restored by photostimulation in the presence of AMPAergic blockade. Video of background-subtracted calcium transients (Rhod3 calcium indicator) in real time. First section shows calcium transient during a spontaneous network burst. Second section shows same culture, during a photostimulated burst in the absence of drug. The bright flash (single frame) represents the blue light illumination passing through the filter set, followed by the calcium transient associated with the network burst once the illuminating light is terminated. Third section shows the same culture after CNQX is added to block AMPAergic transmission. Here photostimulation produces similar burst-associated calcium transient as observed in control conditions.

Supplementary Movie 2 Somatic calcium transients associated with network bursts are eliminated by TTX, but asynchronous transients are observed in TTX and TTX+CTZ. Video of background-subtracted calcium transients (Rhod3 calcium indicator) in real time. Video shows the same culture in control conditions (network burst is shown), then after adding TTX, then with TTX and CTZ. One small slow single calcium transient can be seen in TTX, and then more frequent transients are observed after CTZ is added.

## References

[b1] Rich M. M. & Wenner P. Sensing and expressing homeostatic synaptic plasticity. Trends Neurosci. 30, 119–125 (2007) . 17267052 10.1016/j.tins.2007.01.004

[b2] Davis G. W. Homeostatic signaling and the stabilization of neural function. Neuron 80, 718–728 (2013) . 24183022 10.1016/j.neuron.2013.09.044 PMC3856728

[b3] Wissel J., Manack A. & Brainin M. Toward an epidemiology of poststroke spasticity. Neurology 80, S13–S19 (2013) . 10.1212/WNL.0b013e3182762448 23319481

[b4] Herman S. T. Epilepsy after brain insult: targeting epileptogenesis. Neurology 59, S21–S26 (2002) . 10.1212/wnl.59.9_suppl_5.s21 12428028

[b5] Nielsen J. B., Crone C. & Hultborn H. The spinal pathophysiology of spasticity--from a basic science point of view. Acta Physiol. (Oxf) 189, 171–180 (2007) . 17250567 10.1111/j.1748-1716.2006.01652.x

[b6] Coull J. A. . Trans-synaptic shift in anion gradient in spinal lamina I neurons as a mechanism of neuropathic pain. Nature 424, 938–942 (2003) . 12931188 10.1038/nature01868

[b7] Boulenguez P. . Down-regulation of the potassium-chloride cotransporter KCC2 contributes to spasticity after spinal cord injury. Nat. Med. 16, 302–307 (2010) . 20190766 10.1038/nm.2107

[b8] Marder E. & Goaillard J. M. Variability, compensation and homeostasis in neuron and network function. Nat. Rev. Neurosci. 7, 563–574 (2006) . 16791145 10.1038/nrn1949

[b9] Davis G. W. Homeostatic control of neural activity: from phenomenology to molecular design. Annu. Rev. Neurosci. 29, 307–323 (2006) . 16776588 10.1146/annurev.neuro.28.061604.135751

[b10] Turrigiano G. Homeostatic synaptic plasticity: local and global mechanisms for stabilizing neuronal function. Cold Spring Harb. Perspect. Biol. 4, a005736 (2012) . 22086977 10.1101/cshperspect.a005736 PMC3249629

[b11] Turrigiano G. G., Leslie K. R., Desai N. S., Rutherford L. C. & Nelson S. B. Activity-dependent scaling of quantal amplitude in neocortical neurons. Nature 391, 892–896 (1998) . 9495341 10.1038/36103

[b12] Burrone J., O’Byrne M. & Murthy V. N. Multiple forms of synaptic plasticity triggered by selective suppression of activity in individual neurons. Nature 420, 414–418 (2002) . 12459783 10.1038/nature01242

[b13] Stellwagen D. & Malenka R. C. Synaptic scaling mediated by glial TNF-alpha. Nature 440, 1054–1059 (2006) . 16547515 10.1038/nature04671

[b14] O’Brien R. J. . Activity-dependent modulation of synaptic AMPA receptor accumulation. Neuron 21, 1067–1078 (1998) . 9856462 10.1016/s0896-6273(00)80624-8

[b15] Jakawich S. K. . Local presynaptic activity gates homeostatic changes in presynaptic function driven by dendritic BDNF synthesis. Neuron 68, 1143–1158 (2010) . 21172615 10.1016/j.neuron.2010.11.034 PMC3046391

[b16] Ibata K., Sun Q. & Turrigiano G. G. Rapid synaptic scaling induced by changes in postsynaptic firing. Neuron 57, 819–826 (2008) . 18367083 10.1016/j.neuron.2008.02.031

[b17] Pratt K. G. & Aizenman C. D. Homeostatic regulation of intrinsic excitability and synaptic transmission in a developing visual circuit. J. Neurosci. 27, 8268–8277 (2007) . 17670973 10.1523/JNEUROSCI.1738-07.2007 PMC6673059

[b18] Abbott L. F. & Nelson S. B. Synaptic plasticity: taming the beast. Nat. Neurosci. 3 Suppl, 1178–1183 (2000) . 11127835 10.1038/81453

[b19] Turrigiano G. G. & Nelson S. B. Hebb and homeostasis in neuronal plasticity. Curr. Opin. Neurobiol. 10, 358–364 (2000) . 10851171 10.1016/s0959-4388(00)00091-x

[b20] Sutton M. A. . Miniature neurotransmission stabilizes synaptic function via tonic suppression of local dendritic protein synthesis. Cell 125, 785–799 (2006) . 16713568 10.1016/j.cell.2006.03.040

[b21] Beique J. C., Na Y., Kuhl D., Worley P. F. & Huganir R. L. Arc-dependent synapse-specific homeostatic plasticity. Proc. Natl Acad. Sci. USA 108, 816–821 (2011) . 21187403 10.1073/pnas.1017914108 PMC3021034

[b22] Hou Q., Zhang D., Jarzylo L., Huganir R. L. & Man H. Y. Homeostatic regulation of AMPA receptor expression at single hippocampal synapses. Proc. Natl Acad. Sci. USA 105, 775–780 (2008) . 18174334 10.1073/pnas.0706447105 PMC2206612

[b23] Deeg K. E. & Aizenman C. D. Sensory modality-specific homeostatic plasticity in the developing optic tectum. Nat. Neurosci. 14, 548–550 (2011) . 21441922 10.1038/nn.2772 PMC3415229

[b24] Wagenaar D. A., Pine J. & Potter S. M. An extremely rich repertoire of bursting patterns during the development of cortical cultures. BMC Neurosci. 7, 11 (2006) . 16464257 10.1186/1471-2202-7-11 PMC1420316

[b25] van Pelt J., Wolters P. S., Corner M. A., Rutten W. L. & Ramakers G. J. Long-term characterization of firing dynamics of spontaneous bursts in cultured neural networks. IEEE Trans. Biomed. Eng. 51, 2051–2062 (2004) . 15536907 10.1109/TBME.2004.827936

[b26] Opitz T., De Lima A. D. & Voigt T. Spontaneous development of synchronous oscillatory activity during maturation of cortical networks *in vitro*. J. Neurophysiol. 88, 2196–2206 (2002) . 12424261 10.1152/jn.00316.2002

[b27] Corner M. A., van Pelt J., Wolters P. S., Baker R. E. & Nuytinck R. H. Physiological effects of sustained blockade of excitatory synaptic transmission on spontaneously active developing neuronal networks--an inquiry into the reciprocal linkage between intrinsic biorhythms and neuroplasticity in early ontogeny. Neurosci. Biobehav. Rev. 26, 127–185 (2002) . 11856557 10.1016/s0149-7634(01)00062-8

[b28] Chiappalone M. . Networks of neurons coupled to microelectrode arrays: a neuronal sensory system for pharmacological applications. Biosens. Bioelectron. 18, 627–634 (2003) . 12706572 10.1016/s0956-5663(03)00041-1

[b29] Legrand J. C., Darbon P. & Streit J. Contributions of NMDA receptors to network recruitment and rhythm generation in spinal cord cultures. Eur. J. Neurosci. 19, 521–532 (2004) . 14984403 10.1111/j.0953-816x.2003.03143.x

[b30] Nagel G. . Light activation of channelrhodopsin-2 in excitable cells of *Caenorhabditis elegans* triggers rapid behavioral responses. Curr. Biol. 15, 2279–2284 (2005) . 16360690 10.1016/j.cub.2005.11.032

[b31] Newman J. P. . Closed-loop, multichannel experimentation using the open-source neurorighter electrophysiology platform. Front. Neural. Circuits 6, 98 (2013) . 23346047 10.3389/fncir.2012.00098 PMC3548271

[b32] Partin K. M., Patneau D. K., Winters C. A., Mayer M. L. & Buonanno A. Selective modulation of desensitization at AMPA versus kainate receptors by cyclothiazide and concanavalin A. Neuron 11, 1069–1082 (1993) . 7506043 10.1016/0896-6273(93)90220-l

[b33] Diamond J. S. & Jahr C. E. Asynchronous release of synaptic vesicles determines the time course of the AMPA receptor-mediated EPSC. Neuron 15, 1097–1107 (1995) . 7576653 10.1016/0896-6273(95)90098-5

[b34] Oja E. A simplified neuron model as a principal component analyzer. J. Math Biol. 15, 267–273 (1982) . 7153672 10.1007/BF00275687

[b35] Miller K. & MacKay D. The role of constrains in Hebbian learning. Neural. Comput. 6, 100–126 (1994) .

[b36] Turrigiano G. G. The self-tuning neuron: synaptic scaling of excitatory synapses. Cell 135, 422–435 (2008) . 18984155 10.1016/j.cell.2008.10.008 PMC2834419

[b37] Minerbi A. . Long-term relationships between synaptic tenacity, synaptic remodeling, and network activity. PLoS Biol. 7, e1000136 (2009) . 19554080 10.1371/journal.pbio.1000136 PMC2693930

[b38] Murphy T. H., Blatter L. A., Wier W. G. & Baraban J. M. Spontaneous synchronous synaptic calcium transients in cultured cortical neurons. J. Neurosci. 12, 4834–4845 (1992) . 1361198 10.1523/JNEUROSCI.12-12-04834.1992 PMC6575780

[b39] Goold C. P. & Nicoll R. A. Single-cell optogenetic excitation drives homeostatic synaptic depression. Neuron 68, 512–528 (2010) . 21040851 10.1016/j.neuron.2010.09.020 PMC3111089

[b40] Rutherford L. C., Nelson S. B. & Turrigiano G. G. BDNF has opposite effects on the quantal amplitude of pyramidal neuron and interneuron excitatory synapses. Neuron 21, 521–530 (1998) . 9768839 10.1016/s0896-6273(00)80563-2

[b41] Aoto J., Nam C. I., Poon M. M., Ting P. & Chen L. Synaptic signaling by all-trans retinoic acid in homeostatic synaptic plasticity. Neuron 60, 308–320 (2008) . 18957222 10.1016/j.neuron.2008.08.012 PMC2634746

[b42] Garcia-Bereguiain M. A. . *In vivo* synaptic scaling is mediated by GluA2-lacking AMPA receptors in the embryonic spinal cord. J. Neurosci. 33, 6791–6799 (2013) . 23595738 10.1523/JNEUROSCI.4025-12.2013 PMC3661002

[b43] Wang G., Gilbert J. & Man H. Y. AMPA receptor trafficking in homeostatic synaptic plasticity: functional molecules and signaling cascades. Neural. Plast. 2012, 825364 (2012) . 22655210 10.1155/2012/825364 PMC3359728

[b44] Vitureira N., Letellier M. & Goda Y. Homeostatic synaptic plasticity: from single synapses to neural circuits. Curr. Opin. Neurobiol. 22, 516–521 (2012) . 21983330 10.1016/j.conb.2011.09.006 PMC3378479

[b45] Bassani S., Folci A., Zapata J. & Passafaro M. AMPAR trafficking in synapse maturation and plasticity. Cell Mol. Life Sci. 70, 4411–4430 (2013) . 23475111 10.1007/s00018-013-1309-1 PMC11113961

[b46] Cooke S. F. & Bliss T. V. Plasticity in the human central nervous system. Brain 129, 1659–1673 (2006) . 16672292 10.1093/brain/awl082

[b47] Rabinowitch I. & Segev I. Two opposing plasticity mechanisms pulling a single synapse. Trends Neurosci. 31, 377–383 (2008) . 18602704 10.1016/j.tins.2008.05.005

[b48] Jimbo Y., Tateno T. & Robinson H. P. Simultaneous induction of pathway-specific potentiation and depression in networks of cortical neurons. Biophys. J. 76, 670–678 (1999) . 9929472 10.1016/S0006-3495(99)77234-6 PMC1300072

[b49] Potter S. M. & DeMarse T. B. A new approach to neural cell culture for long-term studies. J. Neurosci. Methods 110, 17–24 (2001) . 11564520 10.1016/s0165-0270(01)00412-5

[b50] Blau A., Neumann T., Ziegler C. & Benfenati F. Replica-moulded polydimethylsiloxane culture vessel lids attenuate osmotic drift in long-term cell cultures. J. Biosci. 34, 59–69 (2009) . 19430119 10.1007/s12038-009-0009-3

[b51] Hales C. M., Rolston J. D. & Potter S. M. How to culture, record and stimulate neuronal networks on micro-electrode arrays (MEAs). J. Vis. Exp. 39, e2056 doi:10.3791/2056 (2010) . 10.3791/2056 PMC3152853 20517199

[b52] Rolston J. D., Gross R. E. & Potter S. M. A low-cost multielectrode system for data acquisition enabling real-time closed-loop processing with rapid recovery from stimulation artifacts. Front. Neuroeng. 2, 12 (2009) . 19668698 10.3389/neuro.16.012.2009 PMC2722905

[b53] Edelstein A., Amodaj N., Hoover K., Vale R. & Stuurman N. Computer control of microscopes using microManager. Curr. Protoc. Mol. Biol. Chapter 14, Unit14 20 (2010) . 10.1002/0471142727.mb1420s92 PMC3065365 20890901

