# Supplementary material for: Upward synaptic scaling is dependent on neurotransmission rather than spiking
Source: Nat Commun. 2015 Mar 9;6:6339. doi: 10.1038/ncomms7339 (PMC4355957; doi:10.1038/ncomms7339)
Supplement: Supplementary Figures and References — Supplementary Figures 1-10 and Supplementary References [file ncomms7339-s1.pdf]

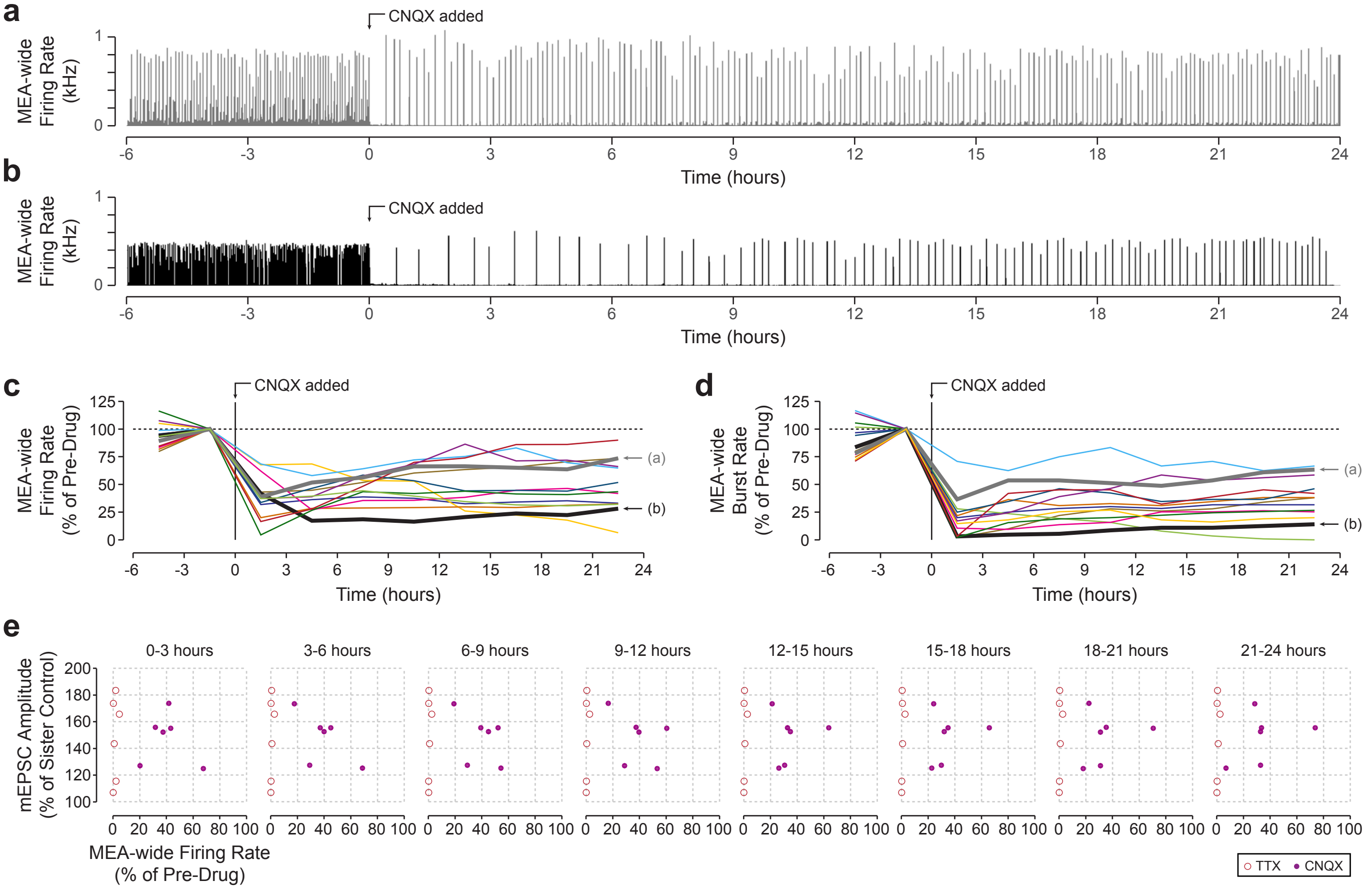

Supplementary Figure 1: AMPAergic transmission blockade moderately reduces firing rate, but the reduction is variable. (a, b) MEA-wide firing rate histograms from 2 recordings before and during application of CNQX (40 µM). While CNQX reduced firing rate, the degree of the reduction varied across cultures. Bin size, 1 s. **(c, d)** MEA-wide firing (c) and burst rate (d) histograms for all 13 CNQX-treated cultures used in this study. Values were normalized to firing rate during 3 hour window before drug/vehicle application. Cultures shown in (a, b) are indicated on each plot. Bin size, 3 h. **(e)** Mean mEPSC amplitude for individual cultures plotted against the average firing rate they exhibited during each 3-hour interval of TTX or CNQX treatment. mEPSC amplitudes were normalized to corresponding sister control cultures, and MEA-recorded activity was normalized to pre-drug levels (from left to right: linear fits, r=-0.0193, -0.1366, -0.0698, -0.0706, 0.0368, 0.0698, 0.0768, 0.1345).

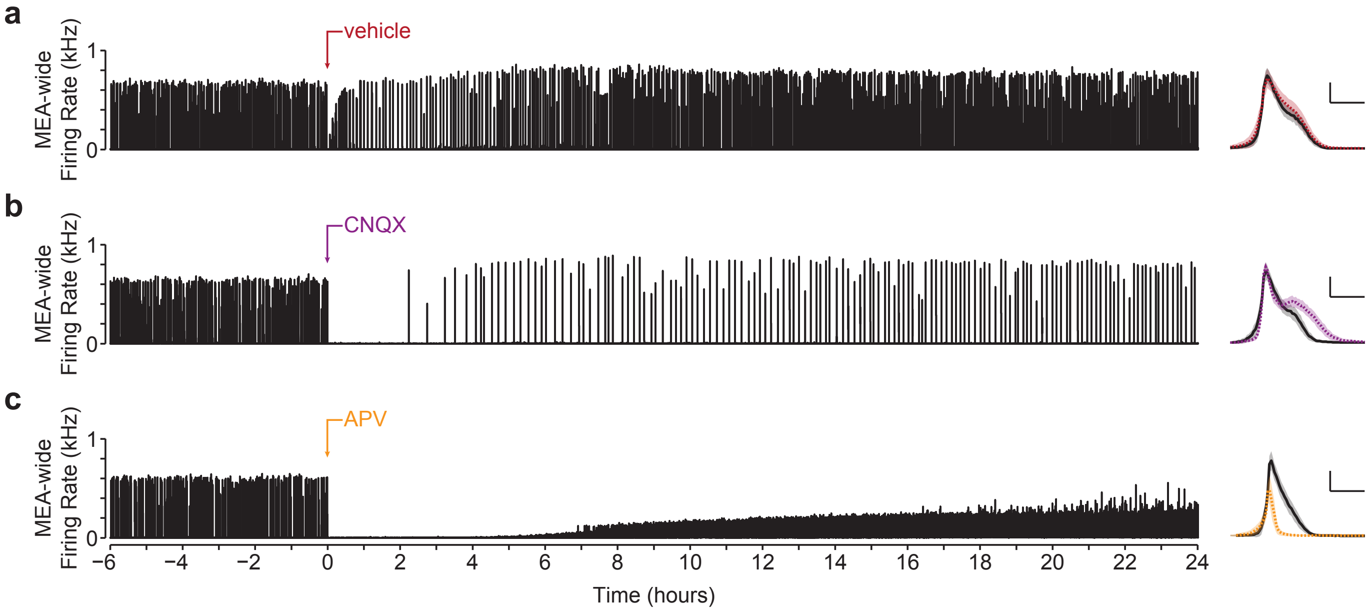

Supplementary Figure 2: NMDAergic transmission is responsible for the late phase of the burst. (a) *Left*, MEA-wide firing rate for a culture treated with vehicle. Bin size, 1 s. Note that reductions in bursting are often observed after media changes or any mechanical disturbance to culture[1]. *Right*, average burst waveform before (black) and after (red) vehicle. Shading denotes s.d. Bin size, 10 ms. Scale bar, 1 kHz, 200 ms. **(b, c)** Same as (a) for cultures treated with 40 µM CNQX (b) or 50 µM APV (c) for 24 hours. When AMPAergic transmission was blocked with CNQX, there was a pronounced increase in burst duration. When NMDAergic transmission was blocked with APV, burst duration was significantly reduced. These results suggest that NMDAergic transmission is responsible for the late phase of the burst. The recordings shown were conducted simultaneously from sister cultures grown on a multi-well MEA (Multichannel Systems, 60-6wellMEA), with each well containing 9 microelectrodes. All other plating and recording procedures were the same as described in Methods.

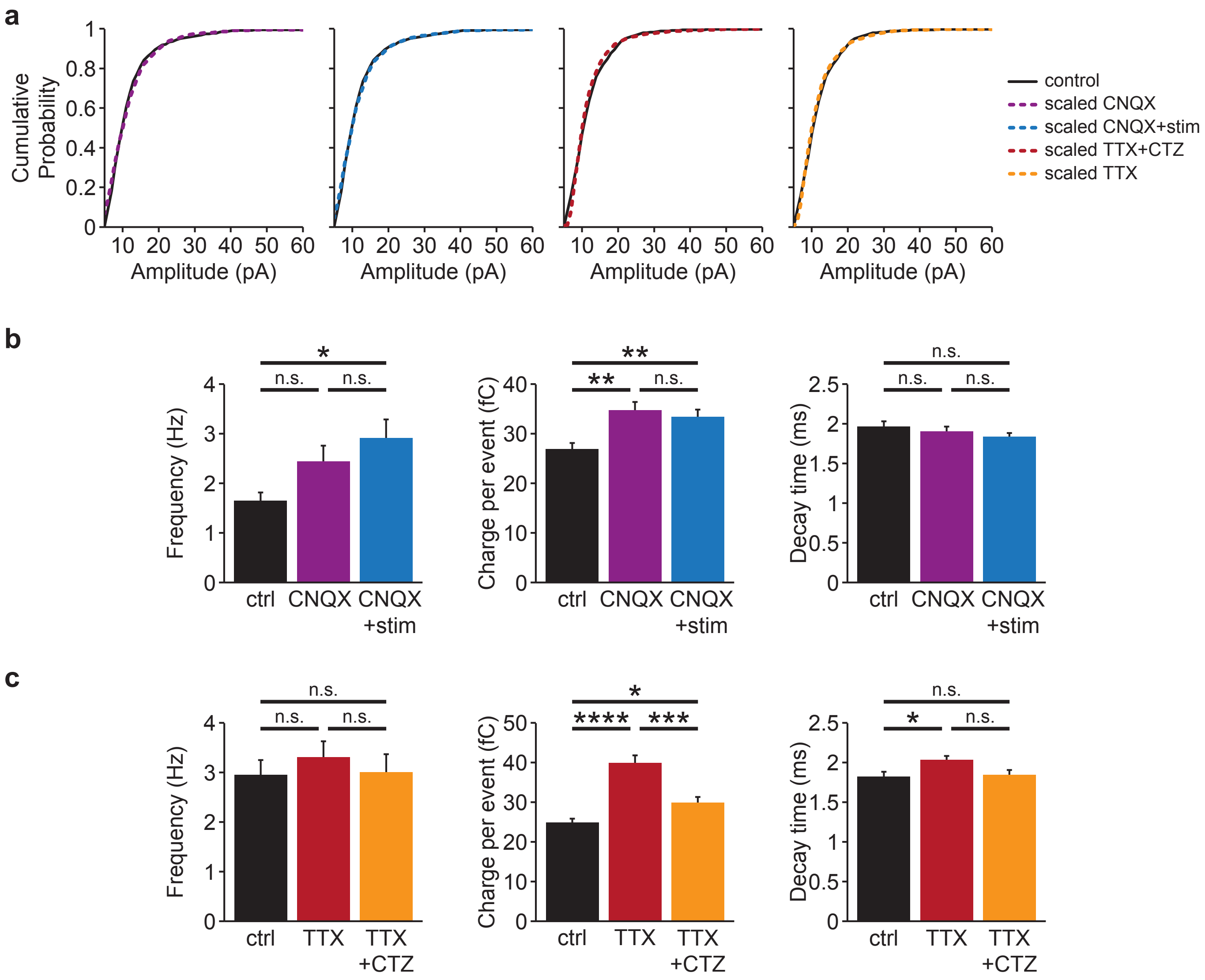

Supplementary Figure 3: mEPSC features following different chronic treatments. **(a)** Scaled mEPSC amplitude distributions for CNQX (40 μM), CNQX+photostimulation, TTX (1 μM), and TTX (1 μM) + CTZ (100 μM; same datasets used for Figs. 6 and 7). Scaled mEPSC distributions for drug-treated cultures were not statistically different from distributions for sister control cultures (CNQX, $p>0.9$; CNQX+stim, $p>0.9$; TTX, $p>0.7$; TTX+CTZ, $p>0.5$). **(b)** Mean frequency (control, 1.6±0.2 pA, n=44 cells; CNQX, 2.3±0.3 pA, n=51 cells; CNQX+photostimulation, 2.6±0.3 pA, n=46 cells; $p<0.02$), charge per event (control, 27.0±1.2 fC; CNQX, 34.8±1.6 fC; CNQX+photostimulation, 33.5±1.4 fC; $p<10^{-3}$), and decay time constant (control, 2.0±0.06 ms; CNQX, 1.9±0.06 ms; CNQX+photostimulation, 1.8±0.04; $p>0.2$) for CNQX+photostimulation experiments. There were significant differences in frequency of control vs. CNQX+photostimulation conditions ($p<10^{-2}$), and in charge per event of control vs. both CNQX cases (control vs. CNQX, $p<10^{-3}$; control vs. CNQX+photostimulation, $p<10^{-3}$). Non-significant differences denoted by n.s. Significant differences denoted by *$p<10^{-2}$, **$p<10^{-3}$. Error bars, s.e.m. **(c)** Mean frequency (control, 3.0±0.3 Hz, n=47 cells; TTX, 3.3±0.4 Hz, n=58 cells; TTX+CTZ, 3.0±0.4 Hz, n=50 cells; $p>0.6$), charge per event (control, 24.9±0.9 fC; TTX, 40.0±1.9 fC; TTX+CTZ, 29.9±1.4 fC; $p<10^{-10}$), and decay time constant (control, 1.8±0.06 ms; TTX, 2.0±0.05 ms; TTX+CTZ, 1.9±0.06 ms; $p<0.02$) for TTX+CTZ experiments. There were significant differences in charge between all conditions (control vs. TTX, $p<10^{-8}$; control vs. TTX+CTZ, $p<10^{-2}$; TTX vs. TTX+CTZ, $p<10^{-4}$), and in decay time constant for cultures treated with TTX (control vs. TTX, $p<10^{-2}$). Non-significant differences denoted by n.s. Significant differences denoted by *$p<10^{-2}$, ***$p<10^{-4}$, and ****$p<10^{-8}$. Error bars, s.e.m.

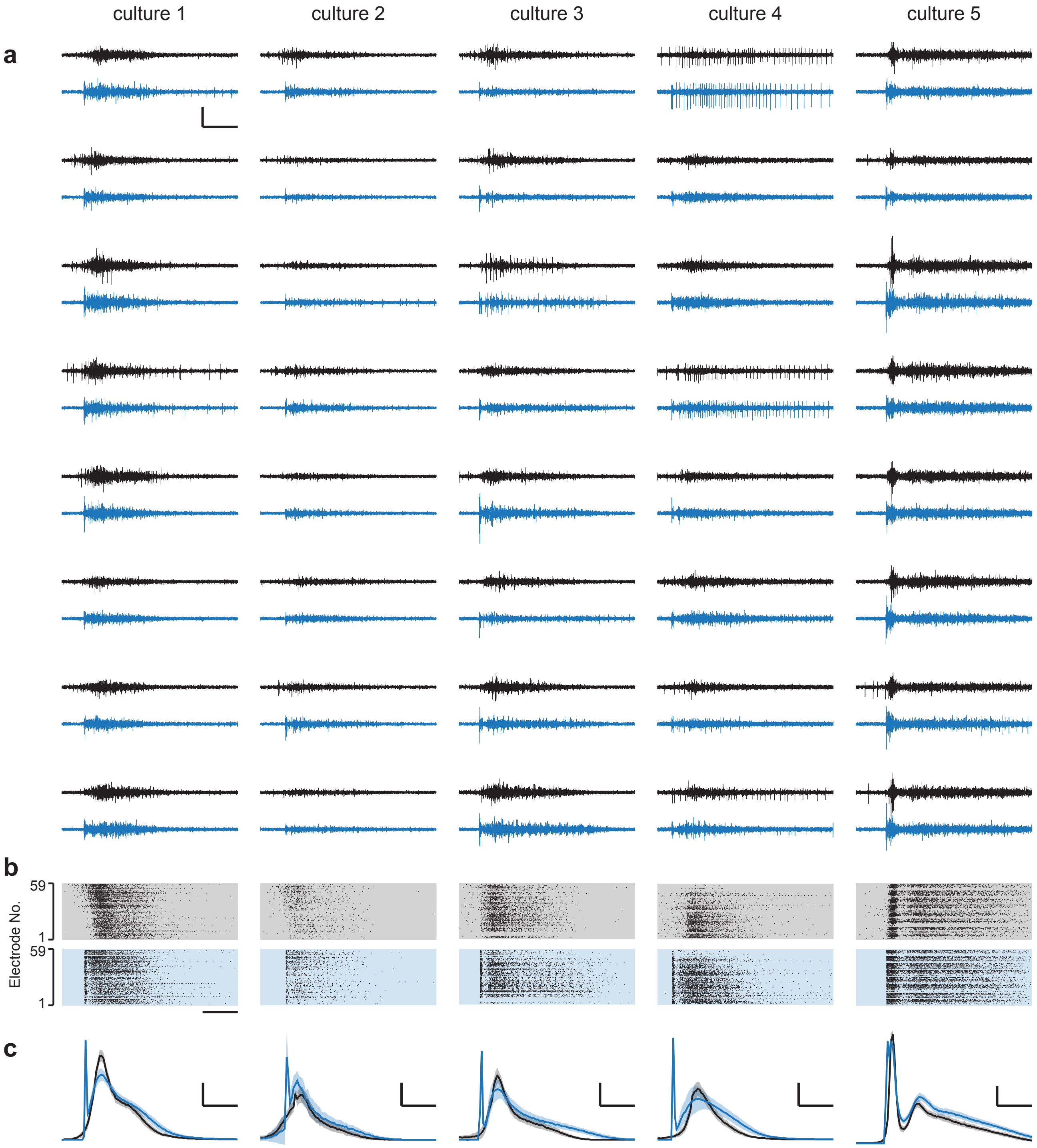

Supplementary Figure 4: Optogenetic stimulation during CNQX treatment effectively mimics spontaneous bursts within individual cultures. **(a)** Raw voltage traces showing spiking activity on individual electrodes during a spontaneous burst before drug application (black), or an optically-evoked burst after the addition of 40 µM CNQX (blue). Data is shown from all 5 chronically-photostimulated cultures with the 8 electrodes that were most active during the pre-drug period selected for display. Scale bars, 100 µV, 200 ms. **(b)** Rastergrams showing spike times for all MEA electrodes corresponding to the bursts shown in (a). The spontaneous burst is shown with a grey background and the optically-evoked burst in the presence of CNQX is shown with a blue background. Scale bar, 200 ms. **(c)** Average MEA-wide firing rate during a burst (spontaneously-occurring, black, 6 hours of burst data; optically-evoked during CNQX, blue, 24 hours of burst data). Shaded regions denote s.d. Bin size, 10 ms. Vertical scale bars, 5 kHz (cultures 1, 3, 4, 5), 2 kHz (culture 2). Horizontal scale bars, 200 ms (all cultures).

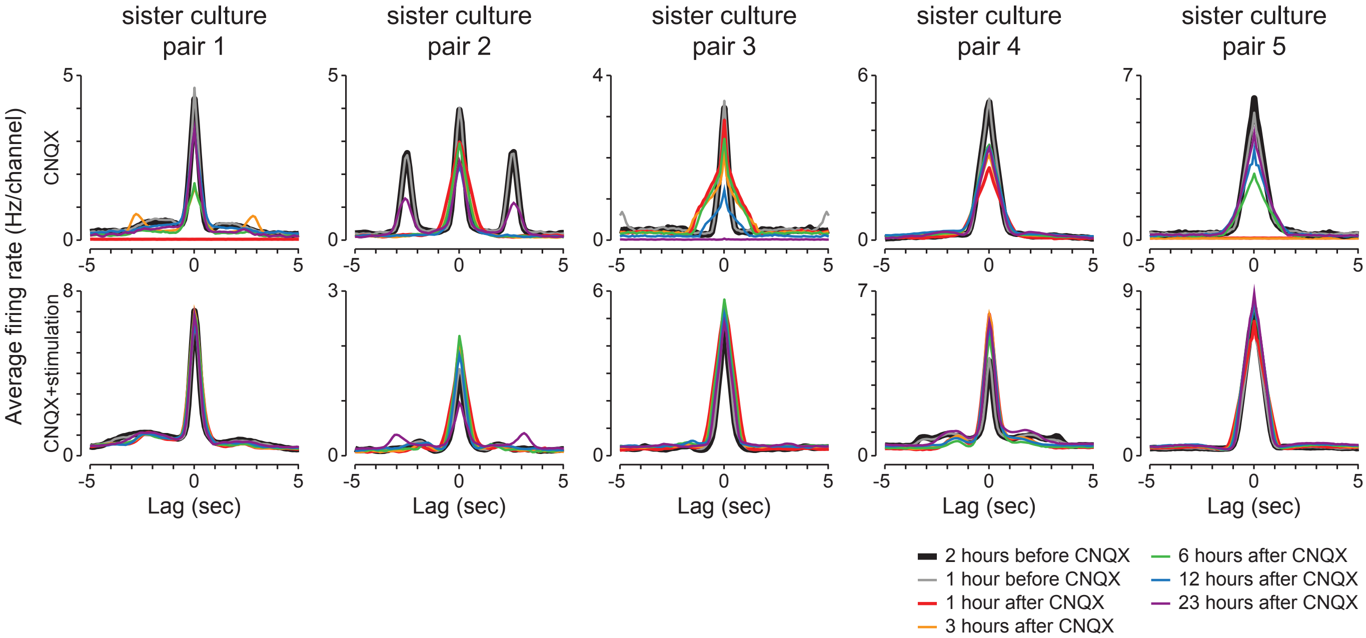

Supplementary Figure 5: Closed-loop optogenetic stimulation during CNQX treatment reproduces channel-to-channel firing correlations. *Top*, the spike detection rate (bin size, 10 ms) cross-correlation was computed for each pair of channels, and averaged across all pairs, for five CNQX-treated cultures (40 µM, no stimulation). This analysis was performed at several time points before and after CNQX was added, denoted by different colored lines. Spiking across channels became less correlated during the first few hours after CNQX application, though spiking correlations generally increased over the 24-hour treatment. *Bottom*, same as top, but for CNQX-treated cultures with optically-restored spiking activity. Closed-loop stimulation maintained pre-drug channel-to-channel firing correlations immediately after CNQX treatment, and this effect was sustained over 24 hours.

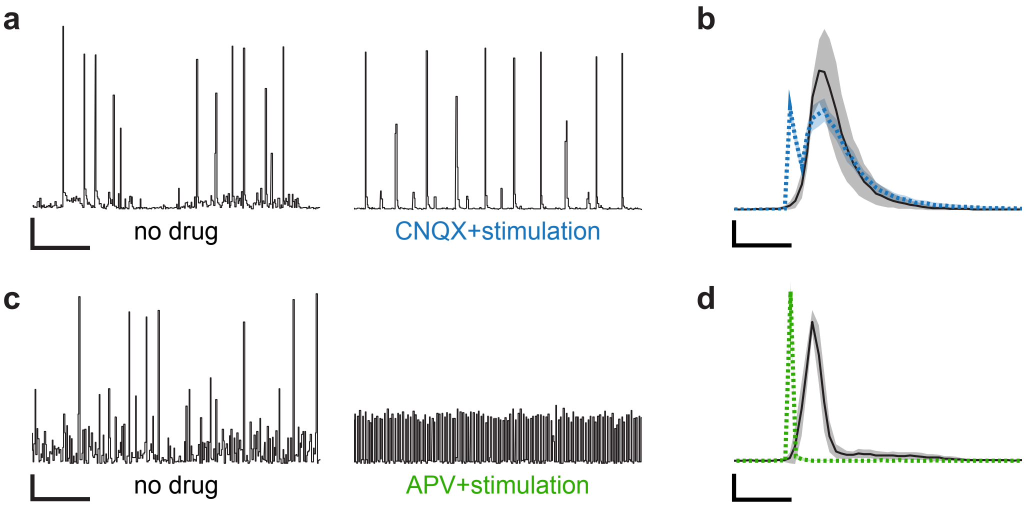

Supplementary Figure 6: NMDAergic transmission facilitates normal bursting during optical stimulation. (a) MEA-wide firing rate for a culture before drug treatment (left), and during CNQX treatment (40 µM) with optogenetically-restored firing rate (right). Bin size, 1 s. Scale bars, 200 Hz, 1 min. **(b)** Average burst waveforms for the two conditions pre-drug (black) and CNQX+photostimulation (blue) conditions. Data used to generate averages was taken for an hour before and after trace shown in (a). Bin size, 10 ms. Scale bars, 2 kHz, 100 ms. **(c)** MEA-wide firing rate for a culture before drug treatment (left), and during APV treatment (50 µM) with optically-restored firing rate (right). Bin size, 1 s. Scale bars, 200 Hz, 1 min. **(d)** Average burst waveforms for the pre-drug (black) and APV+photostimulation (green) conditions. Data used to generate averages was taken for an hour before and after trace shown in (c). Bin size, 10 ms. Scale bars, 800 Hz, 100 ms. Data shown in this figure was generated from cultures infected with AAV2-CaMKIIα-ChR2(H134R)-mCherry, and recordings were performed at 26 DIV (a, b) and 33 DIV (c, d). All other plating, recording, and stimulation parameters were the same as described in Methods.

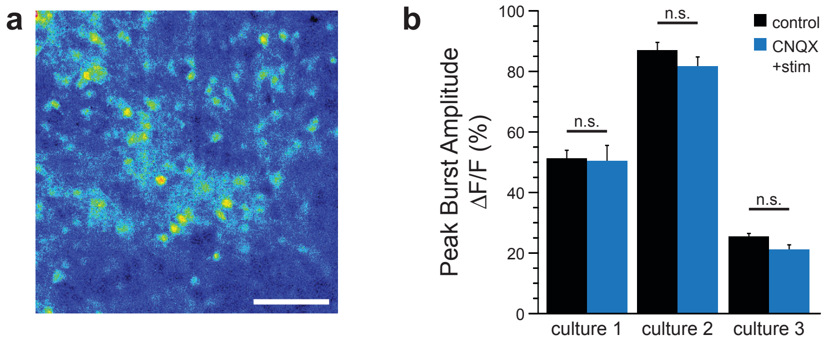

Supplementary Figure 7: Calcium transients are similar for spontaneous and optically-evoked bursts. (a) Sample image of calcium transients from neurons labeled by calcium indicator (Rhod3), which was used to draw regions of interest. Pseudocolored image represents an 8-frame average taken during network burst, which was background subtracted (30 frame average) and median filtered. Scale bar, 100 µm. **(b)** Mean peak response of calcium transients during bursts in 3 different cultures. Calcium transient amplitudes during bursts were not different between spontaneous bursts and optically-evoked bursts in presence of CNQX (p>0.05). Non-significant differences denoted by n.s. Error bars, s.e.m.

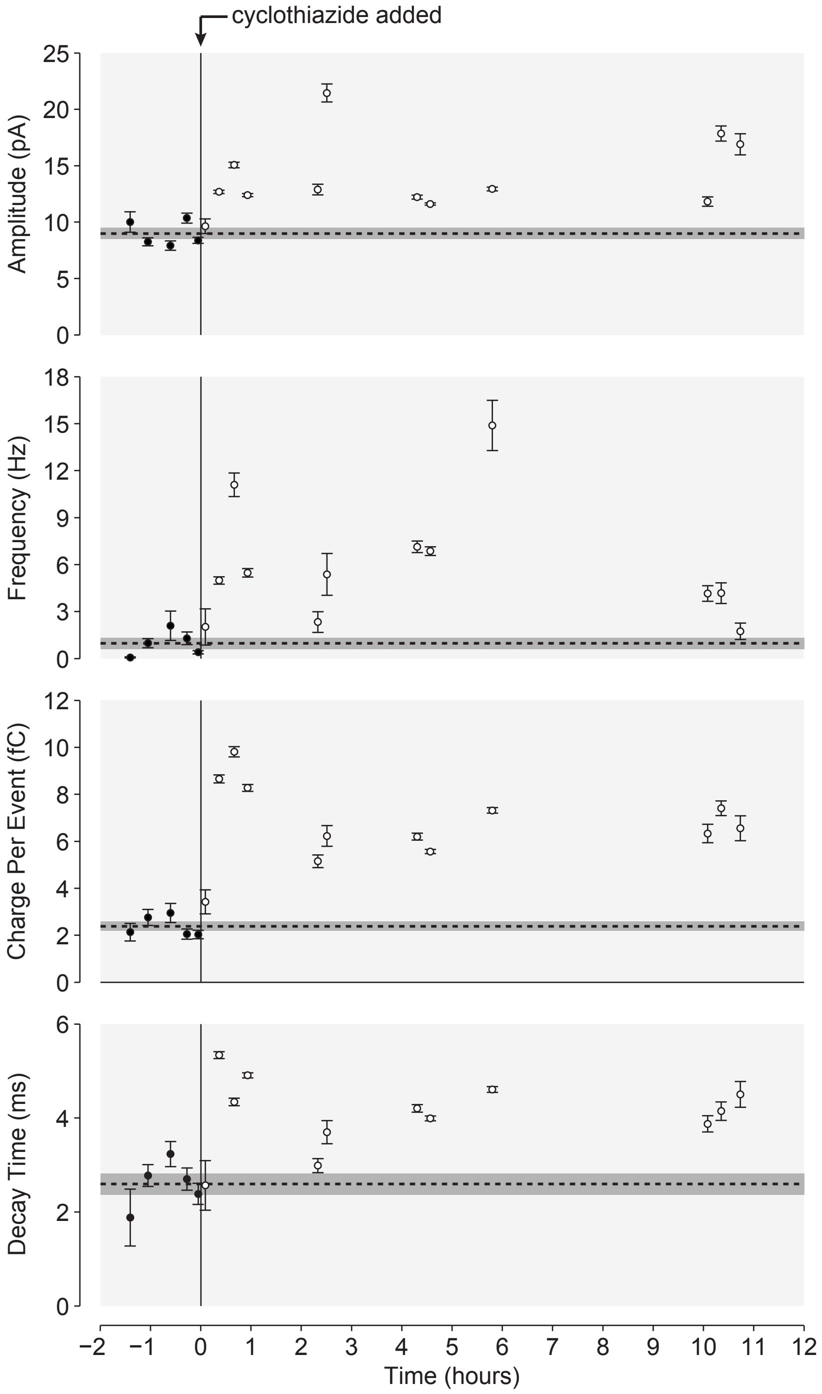

Supplementary Figure 8: Cyclothiazide is effective for enhancing quantal AMPAR activation for at least 11 hours. mEPSCs were recorded from 5 different cells (filled circles), using TTX (1 µM) and bicuculline (20 µM) to isolate AMPAergic events. While recording from the fifth cell, CTZ (100 *µ*M) was added, and mEPSCs were recorded from 11 additional cells (open circles) at various time points over the course of 11 hours. Mean mEPSC amplitude, frequency, charge per event, and decay time constant is shown for all 16 cells. Points just before and after CTZ application represent the same cell. Dotted line denotes the pre-CTZ average of the 5 cell means. Shading and error bars denote s.e.m.

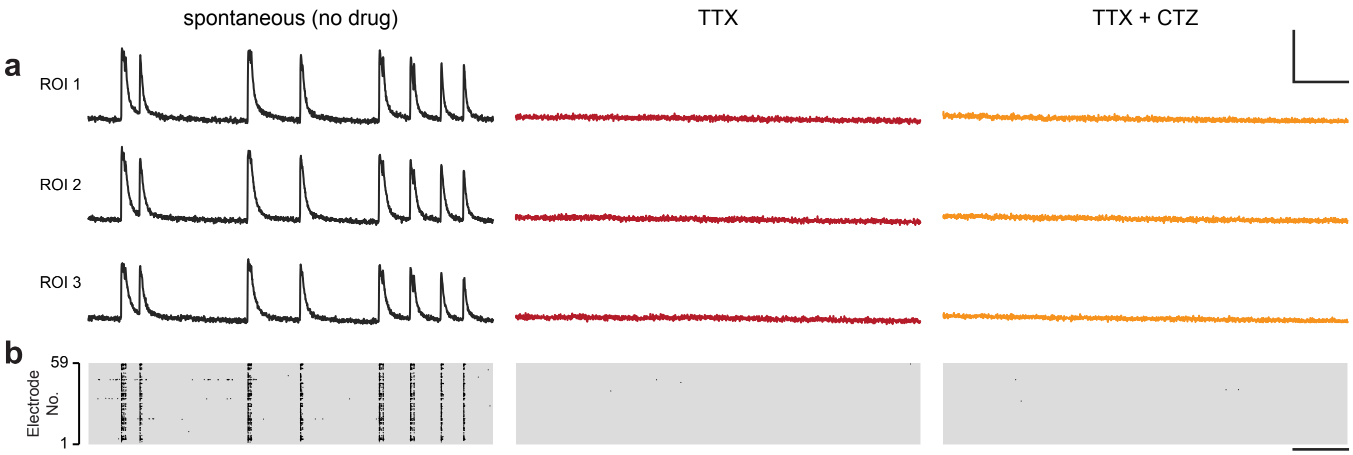

Supplementary Figure 9: Burst-associated calcium transients are eliminated during TTX and CTZ treatment. **(a)** Changes in calcium fluorescence in three example cells/ROIs. High-amplitude, synchronous calcium transients were eliminated upon application of TTX, and later CTZ. Scale bars, 2 s, 40% ΔF/F. **(b)** Rastergrams showing spike times for all MEA electrodes recorded concurrently with calcium transients shown in (a). Scale bar, 2 s. Drug concentrations (in µM): TTX, 1; CTZ, 100.

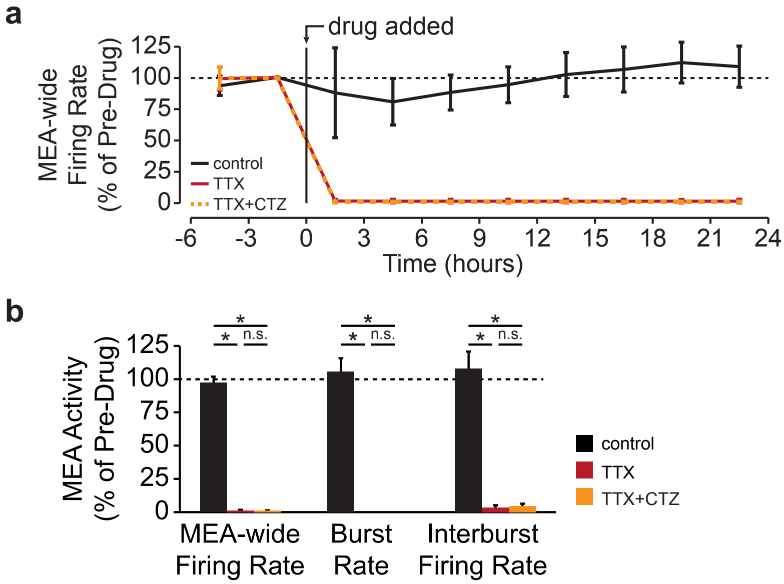

Supplementary Figure 10: Cyclothiazide does not change effects of TTX on spiking activity. (a) Mean MEA-wide firing rate over time for cultures co-treated with TTX and CTZ (n=6 cultures). Control and TTX values from Fig. 2b are shown for comparison (control, n=12 cultures; TTX, n=8 cultures). Spiking was eliminated in all TTX-treated cultures regardless of whether CTZ was present. Bin size, 3 h. Error bars, s.d. **(b)** Mean MEA-wide firing rate, burst rate, and interburst firing rate for the TTX+CTZ condition during the 24-hour treatment window, with control and TTX values from Fig. 2c shown for comparison. TTX+CTZ completely abolished spiking and bursting, and the effect on MEA activity was no different than TTX alone (MEA-wide firing rate, 1.1±0.002%, p>0.3; burst rate, 0%, p=1; interburst firing rate, 4.26±1.91%, p>0.4). Drug concentrations (in µM): TTX, 1; CTZ, 100.

Supplementary References

1. Wagenaar, D.A., Pine, J. & Potter, S.M. An extremely rich repertoire of bursting patterns during the development of cortical cultures. *BMC Neurosci* **7**, 11 (2006).
